# Petri net-based approach to modeling and analysis of selected aspects of the molecular regulation of angiogenesis

**DOI:** 10.1371/journal.pone.0173020

**Published:** 2017-03-02

**Authors:** Dorota Formanowicz, Marcin Radom, Piotr Zawierucha, Piotr Formanowicz

**Affiliations:** 1 Department of Clinical Biochemistry and Laboratory Medicine, Poznan University of Medical Sciences, Grunwaldzka 6, 60-780 Poznań, Poland; 2 Institute of Computing Science, Poznan University of Technology, Piotrowo 2, 60-965 Poznań, Poland; 3 Department of Histology and Embryology, Poznan University of Medical Sciences, Świȩcickiego 6 St., 61-781 Poznań, Poland; 4 Department of Anatomy, Poznan University of Medical Sciences, Świȩcickiego 6, 61-781 Poznań, Poland; 5 Institute of Bioorganic Chemistry, Polish Academy of Sciences, Z. Noskowskiego 12/14, 61-704 Poznań, Poland; King’s College London, UNITED KINGDOM

## Abstract

The functioning of both normal and pathological tissues depends on an adequate supply of oxygen through the blood vessels. A process called angiogenesis, in which new endothelial cells and smooth muscles interact with each other, forming new blood vessels either from the existing ones or from a primary vascular plexus, is particularly important and interesting, due to new therapeutic possibilities it offers. This is a multi-step and very complex process, so an accurate understanding of the underlying mechanisms is a significant task, especially in recent years, with the constantly increasing amount of new data that must be taken into account. A systems approach is necessary for these studies because it is not sufficient to analyze the properties of the building blocks separately and an analysis of the whole network of interactions is essential. This approach is based on building a mathematical model of the system, while the model is expressed in the formal language of a mathematical theory. Recently, the theory of Petri nets was shown to be especially promising for the modeling and analysis of biological phenomena. This analysis, based mainly on t-invariants, has led to a particularly important finding that a direct link (close connection) exist between transforming growth factor *β*1 (TGF-*β*1), endothelial nitric oxide synthase (eNOS), nitric oxide (NO), and hypoxia-inducible factor 1, the molecules that play a crucial roles during angiogenesis. We have shown that TGF-*β*1 may participate in the inhibition of angiogenesis through the upregulation of eNOS expression, which is responsible for catalyzing NO production. The results obtained in the previous studies, concerning the effects of NO on angiogenesis, have not been conclusive, and therefore, our study may contribute to a better understanding of this phenomenon.

## Introduction

The rapidly increasing amount of data concerning the molecular aspects of biological processes led to a conclusion that living organisms represent complex systems, composed of basic building blocks connected by a dense interaction network. The structure of this network determines the structure and functionality of the system. To understand the nature of living organisms, it is not sufficient to analyze the properties of their basic building blocks separately. The studies of the entire network of relations among them are necessary, and the methods suitable for the analysis of complex systems should be applied in these studies [1–3]. Many such methods have already been developed, especially in the area of technical sciences, and some of them can be used for the analyses of biological systems. However, in many cases, the existing methods of systems analysis should be adapted to biological systems, or new methods should be developed.

The basis of systems analysis is a formal model of the studied system, which can be expressed in a formal language of a mathematical theory. Differential equations are frequently used (and not only for the modeling of biological systems), since they represent very powerful mathematical tools. These equations require the precise values of the parameters that correspond to certain quantitative properties of the system, which are often difficult to determine in the case of biological systems, and this makes the construction of a differential equation-based model a difficult (or sometimes impossible) task.

Different approaches have been used for the modeling of biological systems, and especially promising one is based on Petri net theory, whose basic concepts were formulated in the early 1960s by Carl A. Petri, in the context of computer systems [4] (cf. [5–7]). For many years, these types of systems were the main area of application of Petri nets. However, in the mid-1990s, they have been applied for the modeling and analysis of biological phenomena as well, especially the metabolic networks, and this led to further investigations and the development of methods based on Petri nets for the modeling and analysis of complex biological systems [8, 9].

One of the advantages of Petri nets is their intuitive graphical representation, which is very helpful in the phases of the development of a biological system model, and the simulation of system behavior. Petri nets are mathematical objects, therefore they can be analyzed using strict mathematical methods, and many software tools for the simulation and analysis of this type of nets exist.

Building a precise model of a biological system represents a challenging task because such systems are probably among the most complex systems, and, contrary to, for example, the technical ones, they are not constructed by humans. To develop a formal model of a biological system, its structure has to be determined first, which may prove to be difficult.

In our study, the process of the regulation of angiogenesis has been modeled and analyzed. Angiogenesis is a complex physiological process, in which a coordinated sequence of gene expression, protein activity and distribution, as well as cell proliferation and migration, results in the development of new vessels from preexisting ones or from a primary vascular plexus. The development of new vasculature, particularly the formation of new capillaries from endothelial cells, is important in a number of pathological and homeostatic processes. In particular, angiogenesis is a well-described feature of the atherogenic process, in both coronary and carotid disease [10], and this process is necessary for the development and progression of cancer as well. Petri net model of angiogenesis has been already proposed in [11, 12], where the Stochastic Petri net and the reduction techniques have been discussed. These papers focus rather on the simplification of the models that describe complex biological systems and angiogenesis has been seen as a starting point for this concept. The studies presented in these articles relate mainly to VEGF and signaling pathways associated with it. Our research includes a larger number of processes but at a more general level.

## Biological background of angiogenesis

To date, several factors that promote angiogenesis have been described, including physical factors, such as shear stress of blood flow or hypoxia, and biological factors, such as the secretion of various proangiogenic factors. However, hypoxic environment, often associated with tissue inflammation, is considered as the strongest activator of this process and it is among the most studied ones (reviewed in [13, 14]). The regulation of hypoxia-inducible factor 1 (HIF-1) has drawn a particular attention in the modeling of angiogenesis. The regulatory processes differ in the conditions of low oxygen status in cells (hypoxia) and the normal oxygen levels (normoxia).

Human cells require oxygen for the production of the adequate amounts of ATP necessary for metabolic activities, and they have developed the mechanisms of adaptation to hypoxic conditions or oxygen deprivation. Oxygen level changes can lead to the activation or repression of certain homeostatic regulatory genes, allowing the survival of tissues and cells despite the variable environmental conditions. Normal response to ischemia (restriction in blood supply to tissues) encompasses reparative mechanisms summarized by the term neovascularization, which includes 3 processes: angiogenesis, arteriogenesis, and vasculogenesis [15].

All the important compounds and reactions of the analyzed system will be described in the following paragraphs. In the model they will be represented by the so called places and transitions of a Petri net, while here a biological description is given.

The formation of new blood vessels can be divided into four stages, depending on the state of endothelial cells (ECs), which is the main indicator of neovascularization. In the first step, ECs are activated, usually by local hypoxic conditions. The strongest stimulator of hypoxia-driven angiogenesis is HIF-1, which plays an important role in the maintenance of cellular oxygen homeostasis, since it is responsible for the induction of expression of about dozen genes that encode proteins responsible for cell adaptation to hypoxia.

HIF-1 consists of two subunits, *α* and *β*, and HIF family consists of three *α* and three *β* isoforms [16], but we have considered the importance of HIF-1 *α* and *β* isoforms only, and the impact of HIF-2 *α* isoform on erythropoiesis in our model. The role of HIF-3 within the cell remains unclear, and this molecule was not included in the model.

The availability of HIF-1 is mainly determined by the *α* subunit, which is regulated in an oxygen-sensitive manner (controlled by ubiquitin-mediated degradation) under normoxic condition), in contrast to the *β* subunit, which is constitutively expressed [17]. The stability and activity of the *α* subunit is regulated by the post-translational modifications, such as hydroxylation, ubiquitination, acetylation, phosphorylation, and S-nitrosylation.

Prolyl hydroxylase enzymes (PHDs) exert tight control over HIF-1*α* degradation, and their activity depends on the concentration of oxygen. There are three PHD isoforms, PHD1, PHD2, and PHD3 [18], which have the potential to hydroxylate HIF-1*α*. PHD2 was shown to be the key limiting enzyme [19], and we have included only this isoform in our model. In normoxia, proline and asparagine residues of HIF-1*α* are hydroxylated by PHDs and by factor inhibiting HIF (FIH-1), respectively, in an oxygen and Fe ^2+^-dependent manner.

Both Fe(II) and cysteine residues of PHD2 catalytic domain can react with nitric oxide (NO). The most direct mechanism of the modulation of hydroxylase activity by NO is through competition with oxygen for the active-site Fe(II) [20]. NO can inhibit PHD and FIH activity by interacting with the enzyme-bound Fe(II), but this interaction between NO and PHD2 may be much more complex [20].

Hydroxylated HIF-1*α* proline residues serve as markers for the proteasomal degradation of HIF-1*α*, by binding to von Hippel-Lindau tumor suppressor, E3 ubiquitin protein ligase (VHL). VHL is transported from perinuclear granules to the nucleus or cytoplasm by a chaperone protein, VHL binding protein-1 (VBP-1) [21]. Following the VHL binding, HIF-1*α* is polyubiquitinated and rapidly degraded by the 26S-proteasomal system, keeping its expression levels low under normoxic conditions.

The modulation of HIF domains N-TAD and C-TAD is an additional control mechanism of its activity. These domains recruit transcriptional coactivators, such as CREB-binding protein (CBP)/p300 [19]. FIH-1 inhibits the interactions between HIF-1 and its coactivators through the hydroxylation of HIF-1*α* asparagine residues, which serve as a scaffold, linking various transcription factors to basal transcription machinery (BTM), making their role in the activation of HIF-1 especially important [22]. The subsequent binding of HIF-1 to p300/CBP facilitates the adaptation and survival of cells in an environment that changes from normoxia (21% O_2_) to hypoxia (1% O_2_) [19]. During hypoxic conditions, the lack of oxygen leads to strong endothelial activation and inhibits the activity of PHDs, resulting in the suppression of the degradation of *α* subunit, which promotes protein stabilization. Under these conditions, HIF-1*α* migrates from the cytoplasm to nucleus, binding to HIF-1*β* and undergoing dimerization, and forming a transcriptionally active HIF complex.

To date, approximately 100 genes involved in angiogenesis, metabolic adaptation, apoptosis, and metastasis have been identified as direct targets of HIF-1. Some of them, such as erythropoietin (EPO), vascular endothelial growth factor (VEGF), and glucose transporter type 1 (GLUT-1) have been included in our model.

EPO is regulated not only by HIF-1 but also by HIF-2*α* in sirtuin-1 (sirt-1)-dependent manner. The activation of sirt-1 may facilitate HIF-2*α*-directed production of EPO in cellular niches characterized by hypoxic stress, and possibly other environmental stresses [23].

VEGF is the most investigated angiogenic factor, whose activity leads to the production of matrix metalloproteinases (MMPs) and endothelium-derived NO. NO can stabilize and induce the transcriptional activity of HIF-1 through S-nitrosylation, where nitroso group is attached to the sulfur atom of cysteine [24, 25], preventing its degradation and increasing the interaction of HIF-1 complex with its co-activators, p300 and CBP.

NO synthesis in vascular ECs is catalyzed by endothelial nitric oxide synthase (eNOS), which can be activated through the hypoxia-stimulated erythropoiesis (through EPO and EPO receptor (EPOR)), the stimulation of tetrahydrobiopterin (BH_4_), essential cofactor of eNOS in ECs, and the cleavage from calmodulin (CaM) complex in caveolin-1 structures. eNOS is inactivated by CaM dissociation, allowing caveolin-1 to rebind, which is accompanied by renitrosylation of the enzyme and dephosphorylation of eNOS. Increased NO concentration is critical for the following stages of angiogenesis because it induces vessel permeability and the expression of glycolytic genes (GLUT-1) [26].

In the second stage of angiogenesis, MMPs degrade the basement membrane of vessels and extracellular matrix (ECM) components, which allows EC migration and proliferation. ECM represents a scaffold for growth factors that are crucial for the control of angiogenesis, by providing mechanical stability and allowing cell adhesion to ECM. It regulates the key stages of blood vessel morphogenesis and maturation [27].

MMPs may be involved in the recruitment of pericytes as well. The results obtained by Lethi et al. [28] suggest a dominant role of membrane-type 1 matrix metalloproteinase (MT1-MMP) in platelet-derived growth factor receptor-*β* (PDGFR-*β*) axis, leading to the recruitment and migration of mural cells (pericytes and vascular smooth muscles cells (VSMC)). Additionally, transforming growth factor *β*1 (TGF-*β*1) modulates MMPs. Activated TGF-*β*1 binds to the cell-surface type II receptor (TGF-*β*RII), inducing the activation of transmembrane serine/threonine kinases, activin receptor-like kinase-1 (ALK1) and activin receptor-like kinase-5 (ALK5). The activation of ECs is regulated in this way through two opposing pathways. ALK1 induces the signaling pathway that leads to an increase in EC proliferation and migration, while ALK5 inhibits these processes.

Following this, the third stage of angiogenesis is characterized by EC migration and proliferation, through the tip/stalk cell mechanism. During the formation of new vessels, tip cells form a leading head of newly proliferated cells, while stalk cells follow them. Recently, studies have shown that there is continuous competition between the cells of the new outgrowth to become tip cells, through a high expression of vascular endothelial growth factor receptor-2 (VEGFR-2), a major effector of VEGF signaling in angiogenesis, and low expression of vascular endothelial growth factor receptor-1 (VEGFR-1). Through the binding of vascular endothelial growth factor (VEGF) to VEGFR-2 tip cells are able to sense the VEGF gradient and follow it, and this binding promotes EC differentiation, proliferation, and sprouting.

In the last stage of angiogenesis, new vessels are stabilized through the recruitment of mural cells. The interaction between ECs and VSMC-like mural cells is essential for the formation of mature vascular structures. Growing vascular sprouts generate a concentration gradient of platelet-derived growth factor B (PDGFB). High levels of PDGFB in tip cells promote the recruitment of pericytes, which express PDGF receptor *β* (PDGFR-*β*). Biologically active form of PDGF is a dimer consisting of A and B chains. PDGF activity depends on different dimeric forms (AA, AB, or BB). PDGFR is a dimer as well, and it is formed by different combinations of *α* and *β* chains (*α*-*α*, *α*-*β*, *β*-*β*). PDGF-BB and basic fibroblast growth factor (bFGF) have stimulatory effects on ECs. bFGF/fibroblast growth factor receptor (FGFR) signaling pathway plays a critical role in the robust angiogenic response and the upregulation of PDGFR-*α* and PDGFR-*β*, which induce the angiogenic activity of PDGF-BB and PDGF-AB [29]. PDGFR-*β* activity may involve the cooperation with a G-protein coupled receptor for sphingosine-1-phosphate (EDG-1) that binds sphingosine-1-phosphate (SPP), a platelet-derived bioactive sphingolipid secreted by ECs. This induces the production of ECM proteins, promoting the migration of pericytes to the vessel outgrowth [30]. Furthermore, membrane type-1 matrix metalloproteinase (MT1-MMP), the prototypical member of MMP family subset, cooperates with SPP, stimulating EC migration and morphogenic differentiation into capillary-like structures.

Mural cell migration is facilitated by the binding of angiopoietin-1 (Ang-1) on mural cells to endothelial tyrosine kinase receptor 2 (Tie-2 receptor) on the EC surface. Ang-1 induces the expression of heparin-binding epidermal growth factor-like growth factor (HB-EGF) in ECs, and influences the potential of ECs to stimulate VSMC migration, suggesting an indirect mechanism by which Ang-1 recruits VSMCs [31]. Recent studies suggest that the stimulation of EPO leads to an increase in Ang-1 levels, indicating that EPO may regulate angiogenesis, at least partially, by modulating Ang-1 expression.

Angiopoietin-2 (Ang-2), which is produced and stored in Weibel-Palade bodies in ECs, generally functions as an Ang-1 antagonist, and it is upregulated in the hypoxic conditions, in a HIF-dependent manner [32]. Ang-2 is expressed only at the vascular remodeling sites, and plays a crucial role in the destabilization of vessels during normal or pathological angiogenesis [33].

Newly formed vessels are surrounded by the basement membrane, which consists of several types of laminins. It has been hypothesized that interaction between laminins and ECs stops the alterations in actin production, resulting in stationary morphology of ECs and terminating the angiogenic process (reviewed in [27]).

As a summary for the biological explanation of our angiogenesis model, Table 1 containing crucial processes and the literature data is given below.

**Table 1:** Main processes with corresponding literature references.

Process	References
HIF-1 regulatory pathway	[16, 17, 22, 24, 25]
HIF-2 regulatory pathway	[23]
The impact of the factors responsible for the adaptation of the cells to hypoxia	[23, 26, 28, 29, 31–33]

Because of the complex interactions between a large number of molecules, angiogenesis is a complex process, requiring a systems approach to completely understand and elucidate the underlying mechanisms.

In this study, a Petri net-based model of angiogenesis that relies on human cells, is presented and formally analyzed. This model is qualitative, describing the structure of the analyzed system. However, in the case of many biological systems, and angiogenesis as well, the structure is crucial for their functions, making it possible to draw some interesting biological conclusions based on the analysis of the presented model.

## Methods

### Petri nets

Petri nets are mathematical objects, with a structure of a directed bipartite graph *G* = (*V*, *A*). A set of vertices of a graph of this type can be divided into two disjoint subsets, *V*_1_ and *V*_2_, such that ending vertices of each arc (*v*_*i*_, *v*_*j*_) ∈ *A* of this graph belong to different subsets, i.e., *v*_*i*_ ∈ *V*_1_ ∧ *v*_*j*_ ∈ *V*_2_ or *v*_*i*_ ∈ *V*_2_ ∧ *v*_*j*_ ∈ *V*_1_. In Petri nets, vertices that are the elements of one of these subsets are called places, and the ones belonging to the other subset are called transitions. The arcs of this net are labelled by positive integers, called weights.

A directed bipartite graph corresponds to the structure of a Petri net, but this net is not static. One of the fundamental properties of Petri nets is dynamics, which is based on an existence of another net components called tokens. They reside in places and flow from one place to another through transitions (the direction of this flow is determined by the structure of the net, i.e., by the bipartite graph) (compare [5, 9, 34, 35]).

When a Petri net represents a model of a system, places correspond to its passive components, while transitions represent active components, and the flow of tokens models the flow of information through the system. A distribution of tokens over a set of places is called marking, and corresponds to a state of the modeled system.

Formally, a Petri net is a 5-tuple *Q* = (*P*, *T*, *F*, *W*, *M*_0_), where:

*P* = {*p*_1_, *p*_2_,…, *p*_*n*_} is a finite set of places,

*T* = {*t*_1_, *t*_2_,…, *t*_*m*_} is a finite set of transitions,

*F* ⊆ (*P* × *T*) ∪ (*T* × *P*) is a set of arcs,

$W:F\to Z^{+}$ is a weight function,

$M_{0}:P\to N$ is an initial marking,

*P* ∩ *T* = ∅ ∧ *P* ∪ *T* ≠ ∅ [5].

Every transition can have a set of pre-places, i.e., the ones who are its immediate predecessors. Analogously, a transition also can have a set of post-places as its immediate successors. Similarly, every place can have sets of pre- and post-transitions. The flow of tokens through the net is governed by the transition firing rule, where a transition is enabled if the number of tokens residing in every of its pre-places is at least equal to the weight of the arc connecting this place with the transition. An enabled transition can fire, meaning that the tokens can flow from their pre-places to their post-places. The number of tokens flowing between the place and the transition is equal to the weight of the arc connecting these two vertices.

Petri nets are mathematical objects that have very intuitive graphical representation, which facilitates the understanding of the structure and behavior of the net. In this representation, the transitions are shown as rectangles or bars, places as circles, tokens as dots or numbers residing in the places, and arcs as arrows. The arcs are labeled with weights, except when the weight equals one (i.e., usually, the weights equal to one are not explicitly shown in the graphical representation).

This graphical representation of Petri net, although intuitive and useful, is not appropriate for the analysis of net properties. A different representation, called incidence matrix, is used for this purpose. In this matrix, *A* = [*a*_*ij*_]_*n* × *m*_ rows correspond to places and columns correspond to transitions. The matrix entries are integers, and entry *a*_*ij*_, *i* = 1, 2,…,*n*, *j* = 1, 2,…,*m* is equal to the difference between the number of tokens residing in place *p*_*i*_ before and after firing transition *t*_*j*_.

#### t-invariants

If a Petri net is a model of a metabolic network, invariants of the net and an analysis of the relations among them are of particular importance [6, 9, 36–39]. Two kinds of invariants exist here, t-invariants and p-invariants. The former is vector $x\in N^{m}$, which is a solution of the equation

$$\begin{array}{c} A\cdot x=0. \end{array}$$

Similarly, a p-invariant is vector $y\in N^{n}$, representing a solution to the equation

$$\begin{array}{c} A^{T}\cdot y=0. \end{array}$$

Each of these invariants corresponds to a set of transitions or places. More precisely, a support *supp*(*x*) is associated with each t-invariant *x*, representing a set of transitions that correspond to positive entries in *x*, i.e., *supp*(*x*) = {*t*_*j*_ : *x*_*j*_ > 0, *j* = 1, 2,…,*m*}. Every p-invariant *y* has an associated support *supp*(*y*) = {*p*_*i*_ : *y*_*i*_ > 0, *i* = 1, 2,…,*n*}.

Especially important are the minimal invariants, i.e., those whose supports do not contain a support of any other invariant. The consideration of only minimal invariants is sufficient because every invariant can be obtained as a linear combination of the minimal ones. A support of a t-invariant contains transitions whose firing a proper number of times (i.e., the number of times being the invariant entry which corresponds to a given transition) reproduces the marking of a net (i.e., the state of the system modeled by the net does not change). A support of a p-invariant contains places whose weighted sum of tokens is constant (the weights are the positive entries of the invariant).

With the t-invariants a question of their realisability arises [6]. In short, the analysis of t-invariants should base on the *feasible* ones. The existence of non-feasible t-invariants depends on two factors, i.e., insufficient number of tokens in each of the minimal p-invariants in the initial marking and/or read arcs (i.e., arcs going in two directions between a place and a transition). From the further analysis of our model properties it will be clear that such factors are not an issue for the Petri net-based model presented in this paper, therefore all generated t-invariants can be considered as feasible.

Each transition in a Petri net should belong to a support of a feasible t-invariant, i.e., the net should be covered by t-invariants. In that case every modeled elementary biological process contributes to the behavior of the biological system. Every t-invariant corresponds to a subprocess and every transition corresponds to an elementary subprocess. Since every t-invariant has an associated subset of transitions (a support), and the supports can have non-empty intersections, the subprocesses, corresponding to various t-invariants, may be composed of common elementary subprocesses, i.e., modeled by transitions that belong to an intersection of the corresponding supports. Therefore, the analysis of the relations of this type among t-invariants may lead to the elucidation of interactions and dependencies between the subprocesses of the modeled biological system.

#### t-clusters

To find relations among t-invariants, they are grouped into sets called t-clusters [40]. The goal is to acquire different clusters in such a way that they will group together t-invariants modeling subprocesses which possibly interact with each other. On the other hand, the differences between the clusters should be significant enough to distinguish and name such different sets of processes that have been previously identified as t-invariants in the modeled biological system. This is neither a simple nor an automatic process. There are three main “parameters” in this task and changing any one of them will result in a different *clustering*, i.e., a set of clusters for the further biological analysis. These are the distance metric, the linkage (joining) algorithm and the desired number of clusters. In the presented work we have decided to use hierarchical clustering and we have tested different combinations of the above parameters using scripts written in R language, available as S1 Script. Libraries used in our scripts provide different distance metrics and linkage algorithms. The distance metrics are referred to as Binary, Canberra, Pearson’s Correlation, Euclidean, Manhattan, Maximum, Minkowski, and Uncentered Pearson. The clustering algorithms are as follows: Unweighted Pair Group Method with Arithmetic mean (UPGMA), McQuitty linkage, Median linkage (also known as Weighted Pair-Group Method using Centroids, WPGMC), Single linkage, Complete linkage, Centroid linkage (also known as Unweighted Pair-Group Method using Centroids, UPGMC), and Ward’s linkage method [41]. As it has been already stated, the right combination of the distance measure and the clustering method is not an obvious choice. Moreover, the number of clusters is also an important, yet unknown variable. To evaluate the results, the Mean Split Silhouette index (MSS) [42, 43] has been used. Scripts have been prepared in such a way that for each selected distance measure and linkage algorithm, the different clusterings are generated for the selected range of the numbers of clusters, and their content is evaluated using MSS index. The results are presented as PDF files, also for each clustering a dendrogram is generated. Choosing the proper clustering is a challenging task, because the MSS evaluation can help, but alone is not enough. Generally, the most interesting results are obtained by the clustering methods that give the smallest number of single-invariant clusters and the highest MSS value. As it will be discussed later, an initial biological examination of promising clusterings is required, because the evaluation done on the basis of MSS value only may be not sufficient to draw conclusions interesting from the biological point of view.

#### MCT and ADT sets

A support of a t-invariant defines a minimal self-contained subnet, representing a single subprocess within the modeled biological system. All the subprocesses defined by the t-invariants can be analyzed easier if some common subsets of transitions in their supports could be identified. Such subsets are called Maximal Common Transition sets (MCT sets for short), providing an aid in the analysis of biological systems modeled by Petri nets [6, 37]. A set of this type contains transitions which are elements of supports of exactly the same t-invariants. They can be computed using, e.g., a simple algorithm described in [37].

In contrast to t-clusters, which usually induce overlapping subnetworks, MCT sets partition the net into disjunctive subnets and can be considered as the smallest biologically meaningful units [6]. They can have various sizes and also a single transition can form a (trivial) MCT set on its own. For the biological examination of such functional units we consider only non-trivial MCT sets. It should be noted that an MCT set can form a disconnected subnet, i.e., within such a set there can be at least one pair of transitions such that in every path connecting them there is at least one transition not belonging to this set. In [44, 45] different name for such sets has been proposed—maximal Abstract Dependent Transition (ADT) sets. It should be stressed that MCT sets are exactly the same as maximal ADT sets (cf. [6] and [44]). For the latter, a further decomposition has been proposed in [45]—into ADT sets which are not maximal, but induce connected subnets. One of the possible reasons for such a decomposition is to create a hierarchical structure of a net, where connected subnets induced by ADT sets are hidden in the so called macro transitions. Such a hierarchical structure may allow identification of important net components that connect various ADT sets, e.g., interface places for which additional studies can be performed [45]. In our approach we do not use hierarchical net structure, therefore we will use MCT sets. In the presented Petri net based model there is only one MCT set which induces a disconnected subnet and it consists of only two transitions. We have decided its further decomposition would not contribute to the presented analysis.

At the end a difference between t-clusters and MCT sets should be discussed. Both analytical approaches are based on t-invariants. MCT sets consist of transitions, and as it has been already mentioned, they represent a subnet of a net structure, not necessarily a connected one. t-clusters on the other hand group similar t-invariants. The supports of the t-invariants consists of transitions, therefore it is theoretically possible to represent a t-cluster as a multiset of transitions. The same transition appears in such a multiset as many times as it is present in all the supports of t-invariants of a given t-cluster. Such a representation can provide addition help in the understanding of a function of the cluster, because one can highlight a whole region of the net which is involved in all subprocesses which t-invariants of the t-cluster represent. This is just another way of looking on the t-clusters, but it should be noted that such a representation loses the information about the distinct subprocesses represented by the cluster t-invariants. Therefore, assigning a biological function to the t-cluster should first be performed by the analysis of group of its t-invariants. Analysis of the subnet representing the t-cluster can be auxiliary in such an analysis.

#### Knockout analysis

Further analysis has been performed using knockout analysis, which has been previously presented on the model of Duchenne muscular dystrophy [40]. The knockout analysis allows investigating which subprocesses will be influenced (i.e., knocked-out) if some elementary functional unit is disabled. For this task MonaLisa software [46] has been used. Each activity is represented by an MCT set, either a multi-element or a trivial one. Since transitions that belong to the same MCT set always appear together in the same t-invariants, the knockout impact of the members of a given MCT set is always the same. For every activity knocked out, the percent of affected t-invariants have been determined, thus leading to a ranking of the functional units within the model that carry the most and the least impact for the biological system processes.

Such a knockout analysis is based on the structural properties of the model, in this case on the t-invariants. It only gives the information about the percent of all processes that are dependent on the chosen (and disabled) functional unit. In fact, disabling functionality of any given transition should influence the rest of the net in some way that cannot be measured using the described method. Therefore, we have also studied the knockout behavior on the basis of the net dynamic, i.e., by observing tokens fluctuations and changing of transition firing frequencies when some functional units are being disabled. Data describing the normal model behavior (when nothing is disabled) are gathered, they can be compared with the data sets obtained, when a single transition or a non-trivial MCT set has been disabled.

Such a different type of knockout analysis involves a simulation approach. It is performed using token game simulation, in a mode similar to “synchronous” as described in [47], yet in our approach every active transition has 50% chance of firing. Each simulation involves 10,000 steps from the same initial marking and the simulations are repeated 4,000 times. For every step and every repetition data concerning transition firing and tokens distribution are gathered. Finally, a data package is created, containing average firing chance for each transition, as well as the information about the average tokens sum in every place. A standard deviation is calculated for transition firings and for the tokens accumulation from such 4,000 separate simulations to evaluate how repetitive is the behavior of the model. First data package gathered in that way contains the information about the model behavior when nothing is disabled. Then, for every transition a similar simulation procedure is repeated, but this time involving a knockout of that given transition. It means that a selected transition is always considered to be inactive by the simulation engine in every step in every repetition, no matter of the number of tokens in its pre-places. Then the change of firing chances of active transitions, when some important selected transitions, have been marked as knocked out, can be compared.

The net presented in this paper has been created using Snoopy software [48], while the analysis of its formal properties has been performed with INA [49]. Export to INA Petri net format as well as into many other PN file formats is possible and quite easy using Snoopy. Knockout analysis based on t-invariants has been performed using MonaLisa [46]. We have used our own, not yet published tool to acquire data for the described knockout analysis based on token game simulation. Computations involving cluster analysis have been performed using scripts written in R language. They required two additional libraries in R: cluster (v2.0.3) and amap (Another Multidimensional Analysis Package, v0.8.14). Scripts and the t-invariants input file (in fact a simple CSV format) are given in S1 Script.

## Results and discussion

### Analysis of the proposed model

**Fig 1:**
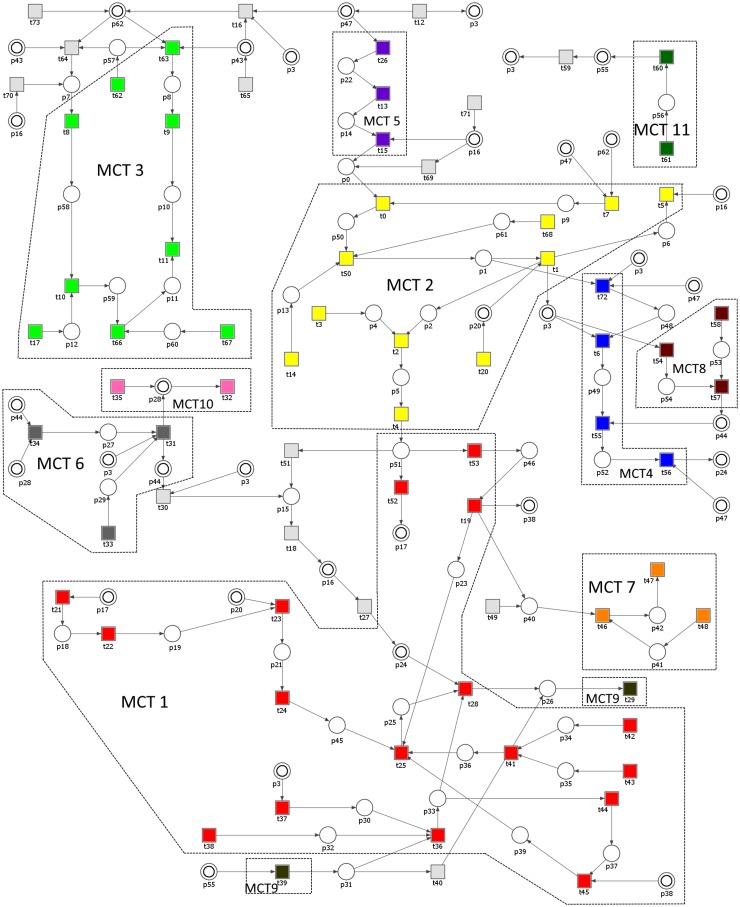
The Petri net model of angiogenesis with marked non-trivial MCT-sets.

The Petri net-based model of the angiogenic process developed and analyzed in this study (shown in Fig 1, also given as S1 File) contains 74 transitions and 63 places. Some places are identically named, but are shown as two concentric circles to improve the model readability, as these are the logical places, representing independent graphical elements that correspond to the same vertex of the net. The names of places and transitions are listed in Tables 2 and 3, respectively. Places correspond to the passive elements of the modeled system, i.e., chemical compounds, reaction products, and substrates, while transitions correspond to the elementary processes occurring in the system.

**Table 2:** The list of places.

Place	Corresponding molecules	Place	Corresponding molecules
*p*_0_	HIF-1*α*-SH stable form not degraded in proteasome	*p*_32_	Tie-2 receptor for Ang-1 and Ang-2
*p*_1_	Active transcriptional factors	*p*_33_	Angiopoietin and Tie receptor complexes
*p*_2_	VEGF	*p*_34_	SPP
*p*_3_	EPO	*p*_35_	EDG-1
*p*_4_	VEGFR-2 on the endothelial surface	*p*_36_	SPP and EDG-1 complex
*p*_5_	VEGF and VEGFR-2 complex	*p*_37_	HB-EGF
*p*_6_	GLUT-1	*p*_38_	EGFR
*p*_7_	PHD-2, activated	*p*_39_	HB-EGF and EGFR complex
*p*_8_	FIH-1, activated	*p*_40_	TGF-1*β*
*p*_9_	HIF-1 *β*, constitutively expressed	*p*_41_	TGF*β*RII
*p*_10_	HIF-1*α*-OH, inactivated	*p*_42_	TGF-1*β*-TGF*β*RII complex
*p*_11_	HIF-1*α*, degraded	*p*_43_	Oxygen
*p*_12_	VHL and VBP-1 complex	*p*_44_	CaM and Ca^2+^ complex
*p*_13_	CBP and p300	*p*_45_	PDGFRs in the absence of ligands
*p*_14_	HIF-1 hydroxylases, inactivated	*p*_46_	MMPs
*p*_15_	eNOS, activated	*p*_47_	Hypoxia
*p*_16_	NO	*p*_48_	EPOR
*p*_17_	bFGF	*p*_49_	EPO and EPOR complex
*p*_18_	FGFRs, activated	*p*_50_	HIF-1*α* and HIF-1 *β* complex, complete (HIF molecule)
*p*_19_	PDGFRs, upregulated	*p*_51_	Tyrosine kinase signaling cascade
*p*_20_	PDGF-AB and PDGF-BB, secreted	*p*_52_	CaM and EPOR complex
*p*_21_	PDGF-AB and PDGF-BB, and PDGFR complex	*p*_53_	CaM free of Ca^2+^
*p*_22_	Endothelium activated by hypoxia	*p*_54_	Increased intracellular Ca^2+^ level
*p*_23_	Recruited pericytes	*p*_55_	HIF-2*α*
*p*_24_	ECs, upon proliferation and migration	*p*_56_	Sirt-1
*p*_25_	Pericytes and smooth muscle cells, upon proliferation and migration	*p*_57_	Fe(II)
*p*_26_	Stable vessels	*p*_58_	HIF-1*α*, hydroxylated (HIF-1*α*-OH-OH)
*p*_27_	CaM-Ca^2+^-caveolin 1 complex	*p*_59_	HIF-1*α*-OH-OH-VHL-VBP1 complex
*p*_28_	Caveolin-1	*p*_60_	Ubiqiutin
*p*_29_	Increased shear stress	*p*_61_	BTM
*p*_30_	Ang-1	*p*_62_	Normoxia
*p*_31_	Ang-2		

**Table 3:** The list of transitions.

Transition	Corresponding reactions	Transition	Corresponding reactions
*t*_0_	HIF-1*α*-SH and HIF-1*β* dimerization	*t*_37_	Constitutive expression of Ang-1
*t*_1_	The genes responsible for cell adaptation to hypoxia	*t*_38_	Tie expression
*t*_2_	VEGF and VEGFR-2 binding	*t*_39_	The expression of angiopoietins
*t*_3_	VEGFR-2 expression	*t*_40_	The remodeling of vessels
*t*_4_	Tyrosine kinase signaling cascade initiation	*t*_41_	The binding of SPP and EDG-1
*t*_5_	GLUT-1 induced processes	*t*_42_	SPP synthesis
*t*_6_	The binding of EPO and EPOR	*t*_43_	EDG-1 synthesis
*t*_7_	Constitutive expression of HIF-1*β*	*t*_44_	The enhancement and increase of synthesis of ECM, and the release of HB-EGF
*t*_8_	Proline hydroxylation	*t*_45_	The binding of HB-EGF and EGFR
*t*_9_	Conservative hydroxylation of asparagine	*t*_46_	The binding of TGF-*β*1 and TGF*β*RII
*t*_10_	The binding of HIF-1-OH-OH and VHL-VBP-1	*t*_47_	ALK-1 or ALK-5 activation
*t*_11_	The lack of HIF-1*α* and HIF-1*β* dimerization	*t*_48_	The expression of TGF*β*RII
*t*_12_	The processes leading to low oxygen concentrations in an organism	*t*_49_	TGF-*β*1 synthesis
*t*_13_	HIF-1 hydroxylase inactivation	*t*_50_	Complex binding to HREs hypoxia responsive elements
*t*_14_	The recruitment of transcriptional coactivators, such as CBP/p300	*t*_51_	The synthesis of factors that stimulate vessel permeability in various ways
*t*_15_	The lack of HIF-1*α* degradation	*t*_52_	The synthesis of factors that stimulate proliferation and survival by bFGF
*t*_16_	The normalization of oxygen status in the organism	*t*_53_	The synthesis of factors that variously stimulate migration through MMPs
*t*_17_	VHL synthesis	*t*_54_	The increase in intracellular Ca^2+^
*t*_18_	NO synthesis	*t*_55_	CaM and EPOR binding
*t*_19_	The recruitment and invasion of pericytes by MMPs	*t*_56_	The upregulation of EPOR signaling pathways
*t*_20_	The secretion of PDGF-AB and PDGF-BB by pericytes	*t*_57_	The binding of CaM and Ca^2+^
*t*_21_	FGFRs activation	*t*_58_	CaM synthesis
*t*_22_	PDGFR upregulation	*t*_59_	HIF-2*α* influence on EPO
*t*_23_	The binding of PDGF-AB and PDGF-BB with the activated PDGFRs	*t*_60_	The influence of sirt-1 on HIF-2*α*
*t*_24_	The removal of ligands	*t*_61_	Sirt-1 synthesis
*t*_25_	Pericyte and smooth muscle cell migration	*t*_62_	The increase of Fe(II)
*t*_26_	Strong endothelial activation	*t*_63_	HIF-1*α* asparagine hydroxylase activation
*t*_27_	Endothelial cells proliferation and migration	*t*_64_	HIF-1*α* proline hydroxylase activation
*t*_28_	The binding process	*t*_65_	Oxygenation
*t*_29_	The use of new stable vessels	*t*_66_	The activation of proteasome degradation
*t*_30_	The activation of eNOS through the interactions with CaM and Ca^2+^ complex	*t*_67_	Ubiquitin synthesis
*t*_31_	The process of cleavage	*t*_68_	The regulation of gene expression through BTM
*t*_32_	Caveolin usage	*t*_69_	S-nitrosylation
*t*_33_	The processes that increase shear stress	*t*_70_	PHD activity influenced by NO
*t*_34_	CaM and caveolin-1 binding	*t*_71_	The processes that lead to the increase in NO
*t*_35_	Caveolin-1 synthesis in ER	*t*_72_	EPOR synthesis induced by hypoxia
*t*_36_	The binding of angiopoietins with Tie receptor on ECs	*t*_73_	Normal cellular state

The presented model is a discrete Petri net with the weights of all arcs being equal to one. The model contains neither information about the speed of reactions, nor substrate or product quantities, as this is a qualitative model, containing only information about the structure of the analyzed system. Functions of a biological system generally depend on its structure, and the analysis of this structure may lead to the deeper understanding of the system properties.

The net is ordinary, meaning that all weights of arcs are equal to one. For each place, all outgoing arcs have the same weight (no quantitative data is considered), so the model is homogeneous. The net is connected but not in the strong sense, i.e., there are undirected paths between any two places, but there may be no directed path between all pairs of places. Connectivity means that there are no independent processes within the model. The net is not structurally conflict-free because it contains places with two or more outgoing arcs (for instance transitions *t*_7_, *t*_63_ and *t*_64_ have the same preceding place *p*_62_). Additionally, the model is pure, i.e., it does not contain any pairs of oppositely directed arcs (read arcs). There are no p-invariants, while there are 48 minimal t-invariants, which cover the net.

When modeling a biological system, the most important part of the analysis is based on t-invariants, and the full list of these invariants is given in Table 4. MCT sets have been determined based on t-invariants and they are listed in Table 5, together with their biological interpretations. It should be noted that only MCT 9 corresponds to a disconnected subnet. It is however one of the smallest sets (2-element one), therefore it has been decided that its further decomposition into smaller sets (each one in that case would be a trivial, i.e., 1-transition MCT set) would not contribute in a significant way to the model analysis.

**Table 4:** The list of t-invariants. The second and third columns show the elements of the support of t-invariants listed in the first column.

t-invariant	MCT sets	Single transitions
*x*_1_	*m*_10_	
*x*_2_	*m*_7_	*t*_49_
*x*_3_	*m*_9_, *m*_11_	*t*_40_
*x*_4_	*m*_6_, *m*_11_	*t*_59_
*x*_5_	*m*_3_	*t*_64_, *t*_65_, *t*_73_
*x*_6_	*m*_2_, *m*_8_	*t*_12_, *t*_18_, *t*_30_, *t*_51_, *t*_69_, *t*_73_
*x*_7_	*m*_3_	*t*_12_, *t*_16_, *t*_64_, *t*_65_
*x*_8_	*m*_2_, *m*_8_	*t*_12_, *t*_16_, *t*_18_, *t*_30_, *t*_51_, *t*_65_, *t*_69_
*x*_9_	*m*_3_	*t*_65_, *t*_70_, *t*_71_, *t*_73_
*x*_10_	*m*_2_, *m*_6_	*t*_12_, *t*_18_, *t*_51_, *t*_69_, *t*_71_, *t*_73_
*x*_11_	*m*_1_, *m*_2_, *m*_6_, *m*_7_, *m*_9_, *m*_11_	*t*_12_, *t*_27_, *t*_69_, *t*_71_, *t*_73_
*x*_12_	*m*_1_, *m*_2_, *m*_4_, *m*_6_, *m*_7_, *m*_8_, *m*_9_, *m*_11_	*t*_12_, *t*_69_, *t*_71_, *t*_73_
*x*_13_	*m*_2_, *m*_5_, *m*_6_	*t*_12_, *t*_18_, *t*_51_, *t*_71_, *t*_73_
*x*_14_	*m*_1_, *m*_2_, *m*_5_, *m*_6_, *m*_7_, *m*_9_, *m*_11_	*t*_12_, *t*_27_, *t*_71_, *t*_73_
*x*_15_	*m*_1_, *m*_2_, *m*_4_, *m*_5_, *m*_6_, *m*_7_, *m*_8_, *m*_9_, *m*_11_	*t*_12_, *t*_71_, *t*_73_
*x*_16_	*m*_1_, *m*_2_, *m*_7_, *m*_8_, *m*_9_, *m*_11_	*t*_12_, *t*_18_, *t*_27_, *t*_30_, *t*_69_, *t*_71_, *t*_73_
*x*_17_	*m*_1_, *m*_2_, *m*_4_, *m*_7_, *m*_8_, *m*_9_, *m*_11_	*t*_12_, *t*_18_, *t*_30_, *t*_69_, *t*_71_, *t*_73_
*x*_18_	*m*_1_, *m*_2_, *m*_5_, *m*_7_, *m*_8_, *m*_9_, *m*_11_	*t*_12_, *t*_18_, *t*_27_, *t*_30_, *t*_71_, *t*_73_
*x*_19_	*m*_1_, *m*_2_, *m*_4_, *m*_5_, *m*_7_, *m*_8_, *m*_9_, *m*_11_	*t*_12_, *t*_18_, *t*_30_, *t*_71_, *t*_73_
*x*_20_	*m*_3_	*t*_12_, *t*_16_, *t*_65_, *t*_70_, *t*_71_
*x*_21_	*m*_2_, *m*_6_	*t*_12_, *t*_16_, *t*_18_, *t*_51_, *t*_65_, *t*_69_, *t*_71_
*x*_22_	*m*_1_, *m*_2_, *m*_6_, *m*_7_, *m*_9_, *m*_11_	*t*_12_, *t*_16_, *t*_27_, *t*_65_, *t*_69_, *t*_71_
*x*_23_	*m*_1_, *m*_2_, *m*_4_, *m*_6_, *m*_7_, *m*_8_, *m*_9_, *m*_11_	*t*_12_, *t*_16_, *t*_65_, *t*_69_, *t*_71_
*x*_24_	*m*_2_, *m*_5_, *m*_6_	*t*_12_, *t*_16_, *t*_18_, *t*_51_, *t*_65_, *t*_71_
*x*_25_	*m*_1_, *m*_2_, *m*_5_, *m*_6_, *m*_7_, *m*_9_, *m*_11_	*t*_12_, *t*_16_, *t*_27_, *t*_65_, *t*_71_
*x*_26_	*m*_1_, *m*_2_, *m*_4_, *m*_5_, *m*_6_, *m*_7_, *m*_8_, *m*_9_, *m*_11_	*t*_12_, *t*_16_, *t*_65_, *t*_71_
*x*_27_	*m*_1_, *m*_2_, *m*_7_, *m*_8_, *m*_9_, *m*_11_	*t*_12_, *t*_16_, *t*_18_, *t*_27_, *t*_30_, *t*_65_, *t*_69_, *t*_71_
*x*_28_	*m*_1_, *m*_2_, *m*_4_, *m*_7_, *m*_8_, *m*_9_, *m*_11_	*t*_12_, *t*_16_, *t*_18_, *t*_30_, *t*_65_, *t*_69_, *t*_71_
*x*_29_	*m*_1_, *m*_2_, *m*_5_, *m*_7_, *m*_8_, *m*_9_, *m*_11_	*t*_12_, *t*_16_, *t*_18_, *t*_27_, *t*_30_, *t*_65_, *t*_71_
*x*_30_	*m*_1_, *m*_2_, *m*_4_, *m*_5_, *m*_7_, *m*_8_, *m*_9_, *m*_11_	*t*_12_, *t*_16_, *t*_18_, *t*_30_, *t*_65_, *t*_71_
*x*_31_	*m*_3_, *m*_8_, *m*_11_	*t*_18_, *t*_30_, *t*_59_, *t*_65_, *t*_70_, *t*_73_
*x*_32_	*m*_1_, *m*_2_, *m*_7_, *m*_8_, *m*_9_, *m*_11_	*t*_12_, *t*_18_, *t*_27_, *t*_30_, *t*_59_, *t*_69_, *t*_73_
*x*_33_	*m*_1_, *m*_2_, *m*_4_, *m*_7_, *m*_8_, *m*_9_, *m*_11_	*t*_12_, *t*_18_, *t*_30_, *t*_59_, *t*_69_, *t*_73_
*x*_34_	*m*_1_, *m*_2_, *m*_5_, *m*_7_, *m*_8_, *m*_9_, *m*_11_	*t*_12_, *t*_18_, *t*_27_, *t*_30_, *t*_59_, *t*_73_
*x*_35_	*m*_1_, *m*_2_, *m*_4_, *m*_5_, *m*_7_, *m*_8_, *m*_9_, *m*_11_	*t*_12_, *t*_18_, *t*_30_, *t*_59_, *t*_73_
*x*_36_	*m*_3_, *m*_8_, *m*_11_	*t*_12_, *t*_16_, *t*_18_, *t*_30_, *t*_59_, *t*_65_, *t*_70_
*x*_37_	*m*_1_, *m*_2_, *m*_7_, *m*_8_, *m*_9_, *m*_11_	*t*_12_, *t*_16_, *t*_18_, *t*_27_, *t*_30_, *t*_59_, *t*_65_, *t*_69_
*x*_38_	*m*_1_, *m*_2_, *m*_4_, *m*_7_, *m*_8_, *m*_9_, *m*_11_	*t*_12_, *t*_16_, *t*_18_, *t*_30_, *t*_59_, *t*_65_, *t*_69_
*x*_39_	*m*_1_, *m*_2_, *m*_5_, *m*_7_, *m*_8_, *m*_9_, *m*_11_	*t*_12_, *t*_16_, *t*_18_, *t*_27_, *t*_30_, *t*_59_, *t*_65_
*x*_40_	*m*_1_, *m*_2_, *m*_4_, *m*_5_, *m*_7_, *m*_8_, *m*_9_, *m*_11_	*t*_12_, *t*_16_, *t*_18_, *t*_30_, *t*_59_, *t*_65_
*x*_41_	*m*_2_, *m*_3_, *m*_5_, *m*_8_	*t*_12_, *t*_18_, *t*_30_, *t*_51_, *t*_65_, *t*_70_, *t*_73_
*x*_42_	*m*_2_, *m*_5_, *m*_6_, *m*_8_	*t*_12_, *t*_18_, *t*_30_, *t*_51_, *t*_73_
*x*_43_	*m*_1_, *m*_2_, *m*_5_, *m*_7_, *m*_8_, *m*_9_, *m*_11_	*t*_12_, *t*_18_, *t*_27_, *t*_30_, *t*_51_, *t*_73_
*x*_44_	*m*_1_, *m*_2_, *m*_4_, *m*_5_, *m*_7_, *m*_8_, *m*_9_, *m*_11_	*t*_12_, *t*_18_, *t*_30_, *t*_51_, *t*_73_
*x*_45_	*m*_2_, *m*_3_, *m*_5_, *m*_8_	*t*_12_, *t*_16_, *t*_18_, *t*_30_, *t*_51_, *t*_65_, *t*_70_
*x*_46_	*m*_2_, *m*_5_, *m*_6_, *m*_8_	*t*_12_, *t*_16_, *t*_18_, *t*_30_, *t*_51_, *t*_65_
*x*_47_	*m*_1_, *m*_2_, *m*_5_, *m*_7_, *m*_8_, *m*_9_, *m*_11_	*t*_12_, *t*_16_, *t*_18_, *t*_27_, *t*_30_, *t*_51_, *t*_65_
*x*_48_	*m*_1_, *m*_2_, *m*_4_, *m*_5_, *m*_7_, *m*_8_, *m*_9_, *m*_11_	*t*_12_, *t*_16_, *t*_18_, *t*_30_, *t*_51_, *t*_65_

**Table 5:** The list of non-trivial MCT sets.

MCT set	Contained transitions	Biological interpretation
*m*_1_	*t*_19_, *t*_21_, *t*_22_, *t*_23_, *t*_24_, *t*_25_, *t*_28_, *t*_36_, *t*_37_, *t*_38_, *t*_41_, *t*_42_, *t*_43_, *t*_44_, *t*_45_, *t*_52_, *t*_53_	The stimulation of neovascularization caused by the selected factors with mitogenic activity in endothelial cells
*m*_2_	*t*_0_, *t*_1_, *t*_2_, *t*_3_, *t*_4_, *t*_5_, *t*_7_, *t*_14_, *t*_20_, *t*_50_, *t*_68_	The activation of the signaling pathways involving mitogen-activated kinases
*m*_3_	*t*_8_, *t*_9_, *t*_10_, *t*_11_, *t*_17_, *t*_62_, *t*_63_, *t*_66_, *t*_67_	The degradation of HIF-1*α* under normoxic conditions
*m*_4_	*t*_6_, *t*_55_, *t*_56_, *t*_72_	EPO pathway regulation
*m*_5_	*t*_13_, *t*_15_, *t*_26_	HIF-1*α* accumulation under hypoxic conditions
*m*_6_	*t*_31_, *t*_33_, *t*_34_	Shear stress-mediated CaM-caveolin-1 cleavage
*m*_7_	*t*_46_, *t*_47_, *t*_48_	The activation of TGF-*β* signaling pathway
*m*_8_	*t*_54_, *t*_57_, *t*_58_	The activation of eNOS modulated by CaM
*m*_9_	*t*_29_, *t*_39_	The stabilization of new vessels by angiopoietins
*m*_10_	*t*_32_, *t*_35_	The regulation of caveolin-1 expression in caveolae
*m*_11_	*t*_60_, *t*_61_	Sirt-1 dependent HIF-2 regulation

### t-invariants, MCT sets, and clusters-based analyses

The analysis of the model is primarily based on t-invariants. On the basis of these transitions have been grouped into 11 MCT sets, while t-invariants has been grouped into t-clusters. The biological significance of these objects has been determined. t-clusters contain t-invariants corresponding to biological subprocesses that interact with each other in some way and new understanding of biological processes can be obtained by a proper construction of t-clusters and the analysis of their contents. This may lead to the discovery of dependencies among the biological subprocesses. For simplicity, in further text, we will sometimes write that a t-invariant contains some MCT sets or transitions to say that these MCT sets and transitions are elements of a support of this invariant.

**Fig 2:**
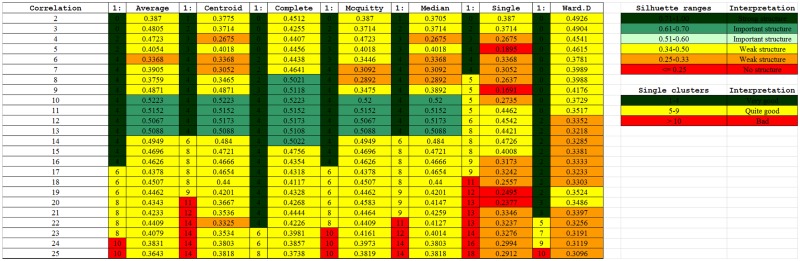
A part of the MSS evaluation table of the Pearson’s Correlation distance and different joining algorithms.

One part of a table that contains the evaluation results of the clustering methods is presented in Fig 2. The complete data are given as an Excel file in S1 Table. Each result is described by two values; the first, narrow column represents the number of single-invariant clusters, while the second column contains the MSS index value for the entire clustering (obtained using a given method for a given number of clusters). The pairs of columns (except the first one) in the Table correspond to the clustering algorithms (linkage methods), while the rows contain the results for given numbers of clusters, for each distance metric.

For the biological analysis 13 clusters has been chosen, obtained using Correlated Person metric and UMPGA (average) linkage method. It requires a further explanation and points out the main issue with the cluster analysis in general. One can see that, for example, for the aforementioned clustering the MSS value is 0.5088, while e.g., for 11 clusters obtained using Binary metric and UPGMA linkage algorithm, MSS value of the entire clustering is higher, i.e., 0.5856 (see S1 Table). However, this clustering contains only seven clusters composed of more than one t-invariant. A detailed analysis of them, shown in Table 6, demonstrates that cluster number 8 contains 28 t-invariants, more than one-half of all t-invariants. This type of cluster corresponds to most of the transitions of the analyzed Petri net and it is very difficult to analyze. In other words, it is quite hard to give such a subset of processes a relevant, common biological function. Therefore, the results presented in Table 6 represent a first step when choosing of the best clustering. The second stage of the analysis, taking into account the composition of each clustering with one of the highest global MSS value is necessary. A careful choosing of distance metric has been also very important step. It seems that correlated Person metric is quite good in measuring the distances for the invariants (i.e., properly distinguishing their similarity) for the clusters computation.

**Table 6:** Two clusterings obtained with different distance metrics and joining algorithms. Mean Split Silhouette (MSS) values are given for every cluster.

Correlation-UPGMA			Binary-UPGMA		
Cluster number	Number of t-invariants	MSS value	Cluster number	Number of t-invariants	MSS value
1	1	0.00	1	1	0.00
2	1	0.00	2	1	0.00
3	1	0.00	3	1	0.00
4	1	0.00	4	1	0.00
5	2	0.38	5	4	0.46
6	10	0.51	6	2	0.56
7	2	0.49	7	2	0.72
8	4	0.64	8	28	0.69
9	8	0.29	9	4	0.33
10	4	0.66	10	2	0.85
11	4	0.59	11	2	0.63
12	2	0.82			
13	8	0.75			

**Fig 3:**
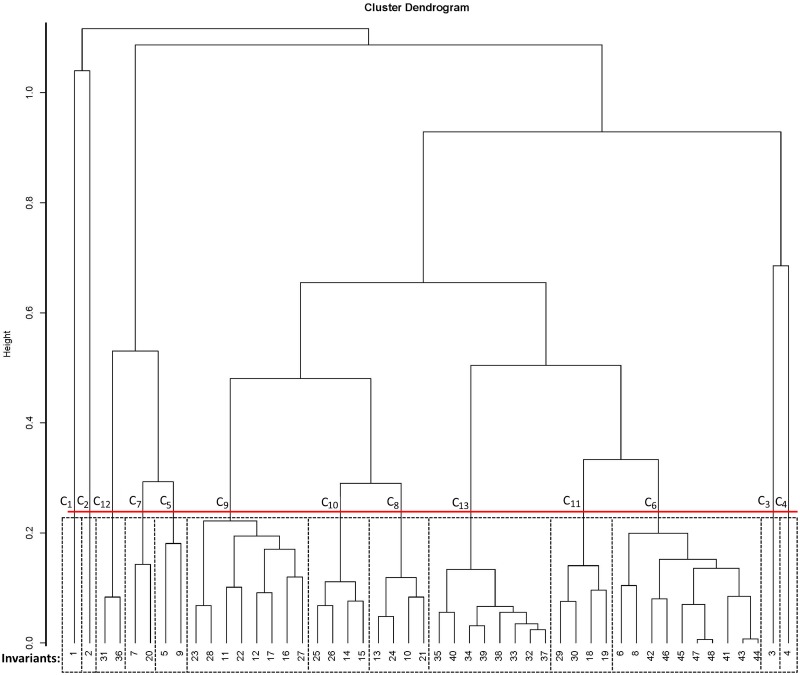
Cluster dendrogram.

Table 7 shows the chosen clusters and their biological interpretation, while Fig 3 represents the dendrogram of the chosen clustering. Red horizontal line represents the threshold level that distinguishes the 13 clusters. The number of t-invariants for each cluster is given below the red line. The obtained clustering served as the basis for the investigations of the relationships between biological subprocesses that correspond to t-invariants.

**Table 7:** The list of clusters.

Cluster	Contained invariants	Biological significance
*c*_1_	*x*_1_	The regulation of caveolin-1 expression caveolae
*c*_2_	*x*_2_	The activation of TGF-*β* signaling pathway
*c*_3_	*x*_3_	The remodeling of vessels and their stabilization, influenced by angiopoietins and HIF-2
*c*_4_	*x*_4_	The regulation of erythropoiesis via HIF-2 and caveolin-1
*c*_5_	*x*_5_, *x*_9_	The degradation of HIF-1*α* under normoxic conditions, influenced by processes that stimulate NO signaling pathway
*c*_6_	*x*_6_, *x*_8_, *x*_41_, *x*_42_, *x*_43_, *x*_44_, *x*_45_, *x*_46_, *x*_47_, *x*_48_	The relationships between metabolic degradation-related pathways and the formation of HIF-1 particles with no effect on remodeling and the regulation of expression of caveolin-1 in caveolae
*c*_7_	*x*_7_, *x*_20_	The degradation of HIF-1*α* under normoxic conditions, influenced by processes that stimulate NO signaling pathway and processes that lead to transition from hypoxia to normoxia, terminating angiogenesis
*c*_8_	*x*_10_, *x*_13_, *x*_21_, *x*_24_	The impact of oxidative stress on the regulation of HIF-1*α* and HIF-1*β* dimerization, together with the formation of active transcriptional factors, responsible for cell adaptation to hypoxia, with particular emphasis on VEGF signaling pathway
*c*_9_	*x*_11_, *x*_12_, *x*_16_, *x*_17_, *x*_22_, *x*_23_, *x*_27_, *x*_28_	The effect of S-nitrosylation on the dimerization of HIF-1*α* molecules, together with the formation of active transcriptional factors responsible for cell adaptation to hypoxia, excluding the influence of the endothelium
*c*_10_	*x*_14_, *x*_15_, *x*_25_, *x*_26_	The effect of S-nitrosylation on the dimerization of HIF-1*α* molecules, together with the formation of active transcriptional factors responsible for cell adaptation to hypoxia
*c*_11_	*x*_18_, *x*_19_, *x*_29_, *x*_30_	The effect of S-nitrosylation on the dimerization of HIF-1*α* molecules, with particular emphasis on TGF_*β*_ signaling pathway, when switching from normoxia to hypoxia
*c*_12_	*x*_31_, *x*_36_	The degradation of HIF-1*α* under normoxic conditions, influenced by CaM-Ca and HIF-2*α*-sirt-1 pathways
*c*_13_	*x*_32_, *x*_33_, *x*_34_, *x*_35_, *x*_37_, *x*_38_, *x*_39_, *x*_40_	The dimerization of HIF-1*α* molecules, together with the formation of active transcriptional factors responsible for cell adaptation to hypoxia, excluding the binding between CaM and ceveolin-1

The analysis of t-invariants showed that the most important process condition affecting almost all of the subprocesses in the presented model is hypoxia and processes that contribute to it are involved in the response to hypoxia. Transition *t*_12_, corresponding to hypoxia, was present in 42 of 48 t-invariants. Six other t-invariants were related either to the regulatory processes, such as the: regulation of eNOS via caveolae (*x*_1_), regulation of the state of endothelium via TGF-*β*1 (*x*_2_), regulation of vascular remodeling by Ang-2 (*x*_3_), regulation of the vascular shear stress response via caveolin-1 and sirt-1 (*x*_4_), or to processes that occur under normoxic conditions, such as the degradation of HIF-1*α* by the ubiquitin-proteasome system (*x*_5_, and the regulation of HIF-1*α* via by NO (*x*_31_)). The last t-invariant, i.e., *x*_31_, corresponds to subprocess comprising phenomena such as proteosomal degradation of HIF-1*α* in normoxia, EPO-mediated activation of eNOS, the increase in cellular calcium levels influenced by EPO, and the pathway sirt-1/HIF-2*α*/EPO pathway. These phenomena are found in one t-invariant; hence, they are closely related to each other. What is especially interesting is that they reflect the crosstalk between NO and HIF signaling pathways. These results are reflected in the content of cluster *c*_12_, which is formed from two t-invariants, i.e., *x*_31_ and *x*_36_, which links HIF and sirt-1 together. By demonstrating that sirt-1 can regulate the activity of HIF proteins, this cluster links HIF and sirt-1 together.

The results of the studies that investigated whether there is a direct interaction between HIF-1*α* and sirt-1 have been conflicting. Dioum et al. [23] have reported that sirt-1 does not target HIF-1*α*, but it deacetylates HIF-2*α* and their interaction promotes HIF-2 transcriptional activity. Different studies suggest that sirt-1 positively regulates the transcriptional activity of HIF-1 [50]. Our results indicate that there is a relationship between HIF-1*α* and sirt-1, and HIF-2*α* and sirt-1.

Forty-two of the remaining t-invariants include subprocesses in which one transition always corresponds to hypoxia. Analysis of these t-invariants showed that none of the supports of t-invariants contain a pair of MCT sets *m*_3_ (degradation of HIF-1*α* under normoxic conditions) and *m*_10_ (regulation of the expression of caveolin-1 in caveolae). This is consistent with the results obtained in the previous studies. Recent studies have highlighted the significance of caveolin-1-dependent control of eNOS, stressing the relationship between these two proteins [51]. Under the baseline conditions, caveolin-1 binds eNOS and inhibits the production of NO through the caveolin-1 scaffolding domain. This implies that the association of eNOS with caveolin-1 maintains eNOS in an inactive state, occupying the calcium/calmodulin binding site of eNOS. Our model showed that, under the normoxic conditions, chronic exposure to NO destabilizes HIF-1*α* and promotes its proteosomal degradation. Therefore, a pair of MCT sets *m*_3_ and *m*_10_ existing side by side would suggest that two contradictory processes coexist. In our model, the inconsistencies of this type do not occur, indicating the correctness of the model.

During hypoxia, HIF-1 may be stabilized in several ways and S-nitrosylation (*t*_69_) plays an important role in this process. It is regulated by the inhibitors of the caveolin-1 peptide scaffolding activity toward eNOS. In the supports of many of t-invariants, S-nitrosylation coincides with the formation of caveolin-1 in hypoxia, showing that the process of HIF-1 stabilization, crucial for cellular adaptation to hypoxic stress, is strictly controlled in mammalian cells to maintain proper oxygen homeostasis.

The analysis of the key phenomena in our model showed that the formation of NO and S-nitrosylation, together with hypoxia, are extremely important processes. The increased levels of NO inhibit angiogenic process [52], but low doses of NO stimulate this process, and this molecule is crucial for the initial steps of angiogenesis. This leads to the conclusion that ECs may have an additional anti-angiogenic mechanism based on NO concentration switch.

Our investigations were able to lead to the identification of the interactions between TGF-*β* signaling pathway activation (*m*_7_), NO synthesis (*t*_18_), the processes that stimulate NO synthesis (*t*_71_), and S-nitrosylation (*t*_69_). These relationships have been determined, among others, in t-invariants *x*_16_, *x*_17_, *x*_27_, and *x*_28_. Additionally, a number of t-invariants contains relationships such as *m*_7_, *t*_18_, and *t*_71_ (*x*_18_, *x*_19_, *x*_29_, *x*_30_) or *m*_7_, *t*_18_, and *t*_69_ (*x*_37_ and *x*_38_). An interesting summary of these observations is cluster *c*_11_, were t-invariants *x*_18_, *x*_19_, *x*_29_, and *x*_30_ coexist. Supports of these invariants contain transitions that correspond to the synthesis of TGF-*β*1, the increase in NO levels, and the reactions of S-nitrosylation, in a condition switching from normoxia to hypoxia. In conclusion, TGF-*β*1 may participate in the inhibition of angiogenesis, through the upregulation of eNOS expression. Furthermore, the analysis of the contents of *x*_7_, *x*_9_, *x*_10_, and *x*_11_ clusters showed that there is a distinct relationship between S-nitrosylation and HIF-1 degradation, leading to a conclusion that NO affects HIF-1 stabilization under hypoxic conditions, which is consistent with the results obtained by Callapina et al. [53].

Additionally, in supports of many t-invariants (*x*_7_, *x*_8_, *x*_20_-*x*_30_, *x*_36_-*x*_41_, *x*_45_-*x*_48_), the transitions that represent opposing processes coexist, e.g., hypoxia (*t*_12_) and oxidation (*t*_65_). This may be considered as a paradox, it may also be explained as an adaptive mechanism of the cells to hypoxic conditions.

Endothelial NOS, a critical regulator of cardiovascular homeostasis, whose dysregulation leads to different vascular pathologies, requires L-arginine and oxygen as substrates for NO synthesis. Our suggestion that, in hypoxic conditions, oxygen demand in the mitochondria of ECs is lower, allowing eNOS to produce higher quantities of NO, which in turn improves vasodilatation and oxygen uptake, may explain the adaptive mechanism of the cells. This can be sufficient only for short-term oxygen flow arrest because a prolonged lack of oxygen leads to cell injury and death. In future, the investigations should focus on how endothelial cells react in normoxia, compared with anoxia (a total depletion of oxygen, an extreme form of hypoxia), rather than hypoxic conditions.

### Knockout analyses

The results of these analyses, sorted according to the importance of the knocked-out MCT set or single transition, are presented in Table 8. This type of knockout is based on the analysis of t-invariants only.

**Table 8:** The impact of net element knockout depending on the affected t-invariants.

MCT-set / transition	Biological function	Affected t-invariants
*t*_12_	The processes leading to low oxygen concentrations in an organism	85.42%
*m*_2_	The activation of the signaling pathway involving mitogen-activated kinases	79.17%
*t*_18_	NO synthesis	66.67%
*m*_8_	The activation of eNOS modulated by CaM	66.67%
*m*_11_	Sirt-1 dependent HIF-2 regulation	66.67%
*m*_9_	The stabilization of new vessels by angiopoietins	60.42%
*m*_7_	The activation of TGF*β* signaling pathway	60.42%
*m*_1_	The stimulation of neovascularization caused by the selected factors with mitogenic activity in endothelial cells	58.33%
*t*_30_	The activation of eNOS through the interactions with CaM and Ca^2+^ complex	58.33%
*t*_65_	Oxygenation	54.17%
*m*_5_	HIF-1*α* accumulation under hypoxic conditions	45.83%
*t*_16_	The normalization of oxygen status in the organism	45.83%
*t*_71_	The processes that lead to the increase in NO	45.83%
*t*_73_	Normal cellular state	45.83%
*t*_69_	S-nitrosylation	33.33%
*m*_6_	Shear stress-mediated CaM-caveolin-1 cleavage	31.25%
*t*_51_	The synthesis of factors that stimulate vessel permeability in various ways	29.17%
*m*_4_	EPO pathway regulation	29.17%
*t*_27_	Endothelial cells proliferation and migration	29.17%
*t*_59_	HIF-2*α* influence on EPO	22.92%
*m*_3_	The degradation of HIF-1*α* under normoxic conditions	16.67%
*t*_70_	PHD activity influenced by NO	12.50%
*t*_64_	HIF-1*α* proline hydroxylase activation	4.17%
*t*_49_	TGF-*β*1 synthesis	2.08%
*m*_10_	The regulation of caveolin-1 expression in caveole	2.08%
*t*_40_	The remodeling of vessels	2.08%

**Fig 4:**
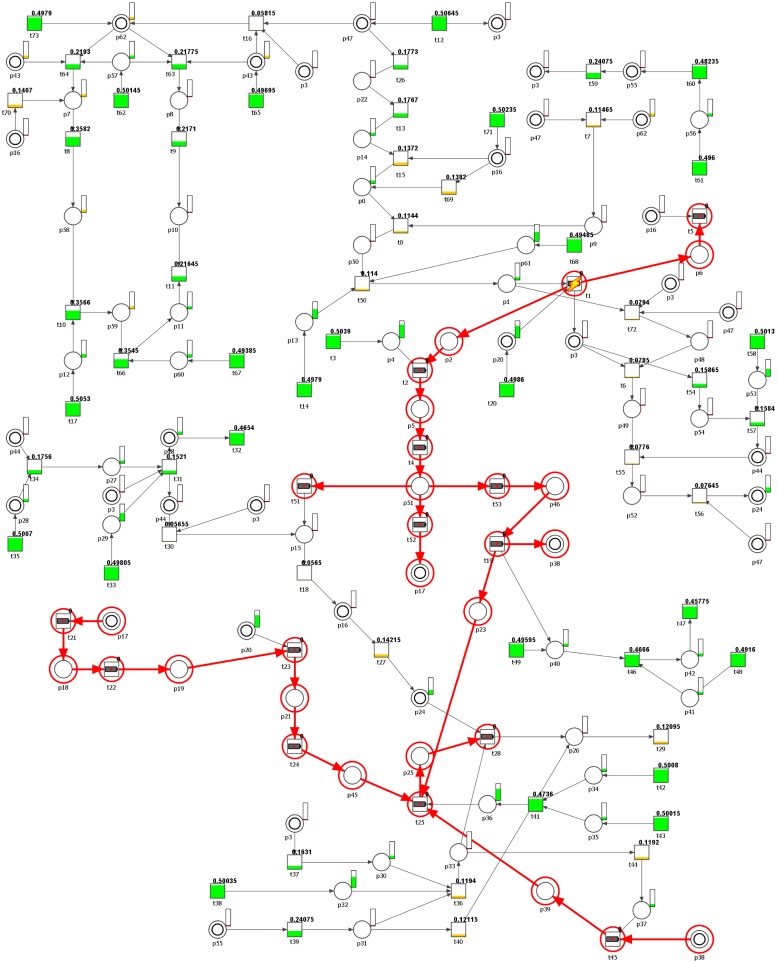
Graphical representation of the knockout results for the entire net, upon the disabling of transition *t*_1_.

**Fig 5:**
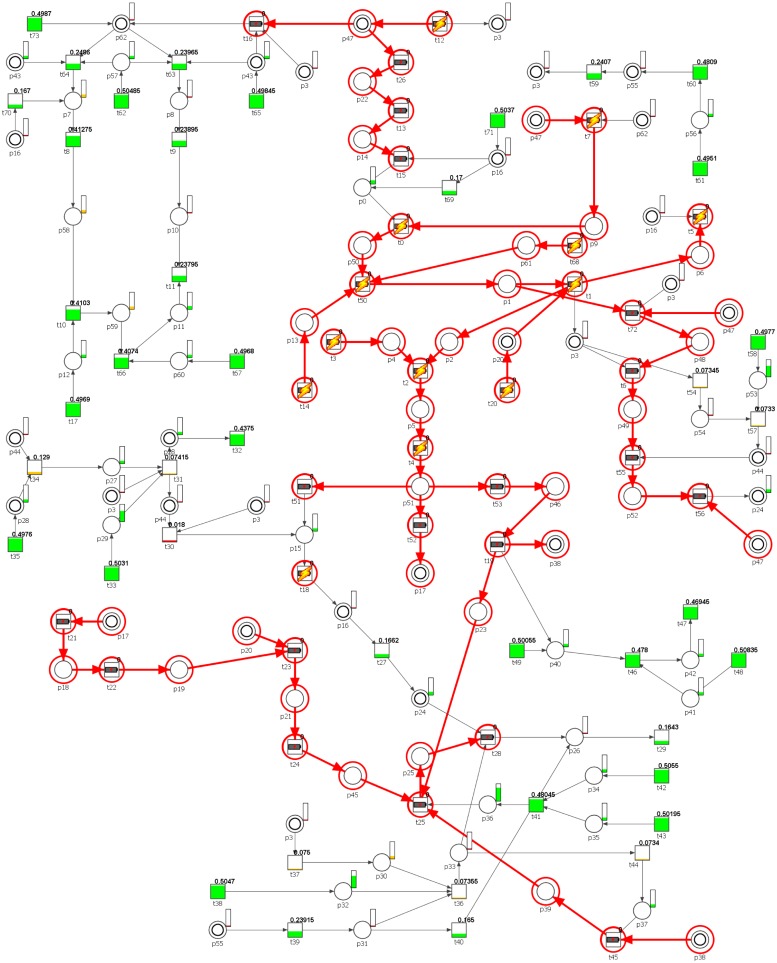
Graphical representation of the knockout results for the entire net, upon the disabling of transitions *t*_12_, *t*_18_, and the entire MCT set *m*_2_.

The second kind of knockout analysis, as described in Methods section, is based on simulation of transition firings in different conditions. The results presented in S2 and S3 Tables have been obtained using our own tool, and they show the impact of manually disabled transitions. The knockout impact of a transition on the net can be presented graphically in the net structure. The graphical representation of the results found in S2 and S3 Tables is presented in Figs 4 and 5, respectively. The transitions with a small thunder icon have been manually disabled (they are considered as permanently inactive by the simulator, as explained earlier), the transitions marked with a red circle (but without the icon) have been knocked out as a result. The colors and a degree of filling of transitions and places represent the average chance of firing for a transition or an average number of tokens accumulated in places during the simulation. For the places this is represented in a separate bars located on the upper right side of each place, while for the transitions there is additional fraction, indicating the average chance of firing. For example, if a graphical representation of a transition is mostly painted green and the fraction is 0.358705, it means that its *average* chance of firing during the simulation is about 35.87%. Yellow and red filings represent a much lower average chance of firing.

The first column in the Tables represents the results obtained following the disabling of transition *t*_1_ (”OFFLINE” status in the second row and the second column of the table). All transitions with the status “KNOCKOUT” have been knocked-out (the firing was completely inhibited) as a direct result of *t*_1_ inability to fire during the simulation. In other words, the offline status means that a transition has been manually set as permanently inactive no matter the tokens in its pre-places, while the knockout status means that a transition could not fire because there were not enough tokens in its pre-places to activate it during all the repeated simulations. The values in the last three columns of the table represent the Reference set, Knockout set, and Difference column, as described in the Methods.

The average chance of firing for many transitions changes depending on the conditions, and for example, transition *t*_0_ had an average chance of firing equal to 14.98% in the Reference set, i.e., in simulations where there had not been any manual disabling of invariants. When *t*_1_ was disabled, *t*_0_ average chance of firing dropped by about 3.82%, leading to the average chance of firing that equaled 11.17% (Knockout set). S3 Table shows the results obtained when *t*_12_, *t*_18_, and the whole MCT set *m*_2_ have been disabled. The word “disabled” in the last column instead of the percent value helps in a quick identification of rows with transitions either being disabled manually (“offline”) or being knocked-out.

Knock-out analyses conclusively show that the most important processes during angiogenesis are the ones leading to lower oxygen concentration (*t*_12_), the activation of the signaling pathway involving mitogen-activated kinases (*m*_2_) and NO synthesis (*t*_18_). The blocking of the transition/MCT set that corresponds to these processes leads to the blockage of 85.42%, 79.17% and 66.67% of all t-invariants in the investigated network, respectively. Since t-invariants correspond to subprocesses involved in angiogenesis, their blockage prevents the formation of new vessels. As previously mentioned, a knockout of an MCT set means a knockout of any transition that represents the element of this set. Therefore, when a knockout of MCT set *m*_2_ influences 79.17% of t-invariants it means that every transition belonging to this set (*t*_0_, *t*_1_, *t*_2_, *t*_3_, *t*_4_, *t*_5_, *t*_7_, *t*_14_, *t*_20_, *t*_50_, and *t*_68_) has the same impact on the net.

Simulation-based knockout analyzes, where transition *t*_1_ or transitions *t*_12_ and *t*_18_, and an MCT set *m*_2_ have been knocked-out (see S2 and S3 Tables) have led to some interesting observations. If transition *t*_1_ is knocked-out, this leads to the reduction of NO synthesis, reduced activation of eNOS and the enhancement of the processes that lead to an increase in EPO levels. Knockout of the transitions that correspond to hypoxia (*t*_12_), NO synthesis (*t*_18_) and activation of the signaling pathway that involves mitogen-activated kinases (*m*_2_), a vast decrease in eNOS activation and processes involving calmodulin and calcium pathways has been observed. Additionally, many transitions were disabled, demonstrating the extent of the influence of the excluded processes on the process of angiogenesis.

### Final summary

A particularly important result of the presented research is finding a direct link (close connection) between molecules, such as TGF-*β*1, eNOS, NO and HIF-1 that play a crucial roles during angiogenesis. We have shown that TGF-*β*1 may participate in the inhibition of angiogenesis through the upregulation of eNOS expression, which is responsible for catalyzing NO production. The results obtained in the previous studies, concerning the effects of NO on angiogenesis, are inconclusive. These studies have suggested that NO inhibits EC migration, which is an essential step in angiogenesis [54]. More recent studies show that NO stimulates angiogenesis in vitro and in vivo, influencing the migration and differentiation of capillary EC. However, the results presented here are simply a starting point for further studies. A wet lab experimental validation of the biological interpretations of mathematical analysis results is necessary. These experiments could provide additional data, which could be used for the improvement of our model. Another direction of the future research should be an extension of this model, through the inclusion of quantitative data. This may be done, for example, by adding weights on the arcs, which correspond to quantities of substances that are involved in the elementary subprocesses occurring in the system. Additionally, a variant of Petri net may be used (e.g., timed, continuous, or hybrid Petri net) to model time dependencies or reaction rates.

## Supporting information

S1 Script Scripts for the R language performing clustering according to the chosen distance, linkage and cluster number. The results are given in PDF files containing MSS evaluation for clusters.
(PDF)

S1 Table The combinations of seven clustering algorithms and eight distance metrics are presented, resulting in different MSS evaluation values for the resulting clusterings. (XLS)

S2 Table Knockout results for all transitions following the disabling of transition *t*_1_. (PDF)

S3 Table Knockout results for all transitions following the disabling of transitions *t*_12_ and *t*_18_, and MCT set *m*_2_. (PDF)

S1 File File in Snoopy representing the discussed net. (SPPED)

## References

[1] Formanowicz P. On the border between biology, mathematics and computer science. BioTechnologia. 2011;92(3):217–220. 10.5114/bta.2011.46536

[2] Klipp E, Liebermeister W, Wierling C, Kowald A, Lehrach H, Herwig R. Systems biology. A textbook. Wiley-VCH, Weinheim; 2009.

[3] Szallasi Z, Stelling J, Perival V. System modeling in cellular biology From concepts to nuts and bolts. The MIT Press Cambridge, Massachusetts; 2006.

[4] Petri CA. In: Communication with automata. vol. 3 of Schriften des IMM. Bonn: Institut für Instrumentelle Mathematik 1962;16–27. (in German).

[5] Murata T. Petri nets: Properties, analysis and applications. Proceedings of IEEE. 1989;90:541–580. 10.1109/5.24143

[6] Sackmann A, Heiner M, Koch I. Application of Petri net based analysis techniques to signal transduction pathway. BMC Bioinformatics. 2006;7:482 10.1186/1471-2105-7-482 17081284 PMC1686943

[7] Reising W. Understanding Petri Nets Modeling Techniques, Analysis Methods, Case Studies. Springer-Verlag, Berlin, Heidelberg 2013.

[8] Matsuno H, Li C, Miyano S. Petri net based descriptions for systematic understanding of biological pathways. IEICE Transactions on Fundamentals of Electronics, Communications and Computer Sciences. 2006;E89A:3166–3174. 10.1093/ietfec/e89-a.11.3166

[9] Koch I, Reisig W, Schreiber F, editors. Modeling in systems biology The Petri net approach. Springer London; 2011.

[10] Moulton KS. Angiogenesis in atherosclerosis: gathering evidence beyond speculation. Current Opinion in Lipidology. 2006;17(5):548–555. 10.1097/01.mol.0000245261.71129.f0 16960504

[11] Napione L, Manini D, Cordero F, Horvath A, Picco A, Pierro MD, et al On the Use of Stochastic Petri Nets in the Analysis of Signal Transduction Pathways for Angiogenesis Process. Lecture Notes in Computer Science. 2009; 5688:281–295. 10.1007/978-3-642-03845-7_19

[12] Cordero F, Horvath A, Manini D, Napione L, Pierro MD, Pavan S, et al Simplification of a complex signal transduction model using invariants and flow equivalent servers. Theoretical Computer Science. 2011; 412(43):6036–6057. 10.1016/j.tcs.2011.06.013

[13] Hirota K, Semenza GL. Regulation of angiogenesis by hypoxia-inducible factor 1. Critical Reviews in Oncology/Hematology. 2006;59(1):15–26. 10.1016/j.critrevonc.2005.12.003 16716598

[14] Krock BL, Skuli N, Simon MC. Hypoxia-induced angiogenesis: good and evil. Genes & Cancer. 2011;2(12):1117–1133. 10.1177/1947601911423654 22866203 PMC3411127

[15] Jaipersad AS, Lip GY, Silverman S, Shantsila E. The role of monocytes in angiogenesis and atherosclerosis. Journal of the Americal College of Cardiology. 2014;63(1):1–11. 10.1016/j.jacc.2013.09.019 24140662

[16] Hendrickson MD, Poyton RO. Crosstalk between nitric oxide and hypoxia-inducible factor signaling pathways: an update. Research and Reports in Biochemistry. 2015;5:147–161.

[17] Hashimoto T, Shibasaki F. Hypoxia-inducible factor as an angiogenic master switch. Frontiers in Pediatrics. 2015;3:33 10.3389/fped.2015.00033 25964891 PMC4408850

[18] Bruick RK, McKnight SL. A conserved family of prolyl-4-hydroxylases that modify HIF. Science. 2001;294(5545):1337–1340. 10.1126/science.1066373 11598268

[19] Ke Q, Costa M. Hypoxia-inducible factor-1 (HIF-1). Molecular Pharmacology. 2006;27(5):1469–1480. 10.1124/mol.106.027029 16887934

[20] Chowdhury R, Flashman E, Mecinović J, Kramer HB, Kessler BM, Frapart YM, et al Studies on the reaction of nitric oxide with the hypoxia-inducible factor prolyl hydroxylase domain 2 (EGLN1). Journal of Molecular Biology. 2011;410(2):268–279. 10.1016/j.jmb.2011.04.075 21601578

[21] Hermann L, Lerman LO, Mukhopadhyay D, Napoli C, Lerman A. Angiogenesis in atherogenesis. Arteriosclerosis, Thrombosis and Vascular Biology. 2006;26(9):1948–1957. 10.1161/01.ATV.0000233387.90257.9b 16794218

[22] Gu J, Milligan J, Huang LE. Molecular mechanisms of hypoxia-inducible factor 1 alpha-p300 interactions. A leucine-rich interface regulated by a single cysteine. The Journal of Biological Chemistry. 2001;276(5):3350–3554. 10.1074/jbc.M009522200 11063749

[23] Dioum EM, Chen R, Alexander MS, Zhang Q, Hogg RT, Gerard RD, et al Regulation of hypoxia-inducible factor 2*α* signaling by the stress-responsive deacetylase sirtuin 1. Science. 2009;324(5932):1289–1293. 10.1126/science.1169956 19498162

[24] Li F, Sonveaux P, Rabbani ZN, Liu S, Yan B, Huang Q, et al Regulation of HIF-1 alpha stability through S-nitrosylation. Molecular Cell. 2007;26:67–74. 10.1016/j.molcel.2007.02.024 17434127 PMC2905600

[25] Yasinska IM, Sumbayev VV. S-nitrosation of Cys-800 of HIF-1*α* protein activates its interaction with p300 and stimulates its transcriptional activity. FEBS Letters. 2003;549(1-3):105–109. 10.1016/S0014-5793(03)00807-X 12914934

[26] Paik JY, Lee K, Ko B, Choe YS, Choi Y, Kim BT. Nitric oxide stimulates 18F-FDG uptake in human endothelial cells through increased hexokinase activity and GLUT1 expression. Journal of Nuclear Medicine. 2005;46(2):365–370. 15695798

[27] Senger DR, Davis GE. Angiogenesis. Cold Spring Harbor perspectives in Biology. 2011;3:a005090 10.1101/cshperspect.a005090 21807843 PMC3140681

[28] Lehti K, Allen E, Birkedal-Hansen H, Holmbeck K, Miyake Y, Chun TH, et al An MT1-MMP-PDGF receptor-*β* axis regulates mural cell investment of the microvasculature. Genes & Development. 2005;19(8):979–991. 10.1101/gad.1294605 15805464 PMC1080136

[29] Raju R, Palpetta SM, Sandhya VK, Sahu A, Alipoor A, Balakrishnan L, et al A network map of FGF-1/FGFR signaling system. Journal of Signal Transduction. 2014;2014:962962 10.1155/2014/962962 24829797 PMC4009234

[30] Jain RK. Molecular regulation of vessel formation. Nature Medicine. 2003;9:685–693. 10.1038/nm0603-685 12778167

[31] Iivanainen E, Nelimarkka L, Elenius V, Heikkinen SM, Junttila TT, Sihombing L, et al Angiopoietin-regulated recruitment of vascular smooth muscle cells by endothelial-derived heparin binding EGF-like growth factor. FASEB Journal. 2003;17(12):1609–16021. 10.1096/fj.02-0939com 12958167

[32] Simon MP, Tournaire R, Pouyssegur J. The angiopoietin-2 gene of endothelial cells is up-regulated in hypoxia by a HIF binding site located in its first intron and by the central factors GATA-2 and Ets-1. Journal of Cellular Physiology. 2008;217(3):809–818. 10.1002/jcp.21558 18720385

[33] Felcht M, Luck R, Schering A, Seidel P, Srivastava K, Hu J, et al Angiopoietin-2 differentially regulates angiogenesis through TIE2 and integrin signaling. The Journal of Clinical Investigation. 2012;122(6):1991–2005. 10.1172/JCI58832 22585576 PMC3366398

[34] David R, Alla H. Discrete, continuous and hybrid Petri nets. Springer Verlag, Berlin Heidelberg; 2010.

[35] Reisig W. Petri nets: an introduction. Springer Verlag, Heidelberg; 1985.

[36] Formanowicz D, Kozak A, Głowacki T, Radom M, Formanowicz P. Hemojuvelin-hepcidin axis modeled and analyzed using Petri nets. Journal of Biomedical Informatics. 2013;46(6):1030–1043. 10.1016/j.jbi.2013.07.013 23954231

[37] Formanowicz D, Sackmann A, Kozak A, Błażewicz J, Formanowicz P. Some aspects of the anemia of chronic disorders modeled and analyzed by petri net based approach. Bioprocess and Biosystems Engineering. 2011;34(5):581–595. 10.1007/s00449-010-0507-6 21221653 PMC3092940

[38] Formanowicz D, Wanic-Kossowska M, Pawliczak E, Radom M, Formanowicz P. Usefulness of serum interleukin-18 in predicting cardiovascular mortality in patients with chronic kidney disease-systems and clinical approach. Scientific Reports. 2015;5:18332 10.1038/srep18332 26669254 PMC4680867

[39] Grafahrend-Belau E, Schreiber F, Heiner M, Sackmann A, Junker BH, et al Modularization of biochemical networks based on classification of Petri net t-invariants. BMC Bioinformatics. 2008;9:90 10.1186/1471-2105-9-90 18257938 PMC2277402

[40] Grunwald S, Speer A, Ackermann J, Koch I. Petri net modelling of gene regulation of the Duchenne muscular dystrophy. Biosystems. 2008;92(2):189–205. 10.1016/j.biosystems.2008.02.005 18372101

[41] Jain AK, Dubes RC. Algorithms for clustering data. Prentice-Hall Inc., New Jersey 1988.

[42] Rousseeuw PJ. Silhouettes: A graphical aid to the interpretation and validation of cluster analysis. Journal of Computational and Applied Mathematics. 1987;20:53–65. 10.1016/0377-0427(87)90125-7

[43] Kaufman L, Rousseeuw PJ. Finding groups in data: An introduction to cluster analysis. John Wiley and Sons, New York 1990.

[44] Winder K. Invariant-supported Petri Net Structuring (in German). Diploma thesis. BTU Cottbus, Department of Computer Science. 2006.

[45] Heiner M, Understanding Network Behaviour by Structured Representations of Transition Invariants-A Petri Net Perspective on Systems and Synthetic Biology Algorithmic Bioprocesses. Springer 2009;367–389.

[46] Einloft J, Ackermann J, Nöthen J, Koch I. MonaLisa-visualization and analysis of functional modules in biochemical networks. Bioinformatics. 2013;29(11):1469–1470. 10.1093/bioinformatics/btt165 23564846

[47] Balazki P, Lindauer K, Einloft J, Ackermann J, Koch I. MONALISA for stochastic simulations of Petri net models of biochemical systems. BMC Bioinformatics. 2015;16:215 10.1186/s12859-015-0596-y 26156221 PMC4496887

[48] Heiner M, Herajy M, Liu F, Rohr C, Schwarick M. Snoopy—a unifying Petri net tool. Application and Theory of Petri nets. Lecture Notes in Computer Science. 2012;7347:398–407. 10.1007/978-3-642-31131-4_22

[49] INA Home page. Available at http://www2.informatik.hu-berlin.de/starke/ina.html.

[50] Laemmle A, Lechleiter A, Roh V, Schwarz C, Portmann S, Furer C, et al Inhibition of SIRT1 impairs the accumulation and transcriptional activity of HIF-1*α* protein under hypoxic conditions. PLoS ONE 2012;7(3):e33433 10.1371/journal.pone.0033433 22479397 PMC3316573

[51] Trane AE, Pavlov D, Sharma A, Saqib U, Lau K, van Petegem F, et al Deciphering the binding of caveolin-1 to client protein endothelial nitric-oxide synthase (eNOS): scaffolding subdomain identification, interaction modeling, and biological significance. The Journal of Biological Chemistry. 2014;289(19):13273–13283. 10.1074/jbc.M113.528695 24648521 PMC4036337

[52] Isenberg JS, Ridnour LA, Perruccio EM, Espey MJ, Wink DA, Roberts DD. Thrombospodin-1 inhibits endothelial cell responses to nitric oxide in a cGMP-dependent manner. Proceedings of the National Academy of Sciences of the United States of America. 2005;102(37):13141–13146. 10.1073/pnas.0502977102 16150726 PMC1201579

[53] Callapina M, Zhou J, Schmid T, Kohl R, Brune B. NO restores HIF-1*α* hydroxylation during hypoxia: Role of reactive oxygen species. Free Radical Biology and Medicine. 2005;39(7):925–936. 10.1016/j.freeradbiomed.2005.05.009 16140212

[54] Goligorsky MS, Budzikowski AS, Tsukahara H, Noiri E. Co-operation between endothelin and nitric oxide in promoting endothelial cell migration and angiogenesis. Clinical and Experimental Pharmacology and Physiology. 1999;26(3):269–271. 10.1046/j.1440-1681.1999.03029.x 10081626

