# Supplementary material for: Petri net-based approach to modeling and analysis of selected aspects of the molecular regulation of angiogenesis
Source: PLoS One. 2017 Mar 2;12(3):e0173020. doi: 10.1371/journal.pone.0173020 (PMC5333880; doi:10.1371/journal.pone.0173020)
Supplement: S1 Script — The results are given in PDF files containing MSS evaluation for clusters. (PDF) [file pone.0173020.s001.pdf]

Script 1:
parameters:

distance_metric: *binary, canberra, euclidean, manhattan, maximum, minkowski*
joining _method: *average, centroid, complete, mcquitty, median, single, ward.D*
path: path for invariants file
invariants_input_file: name of invariants file
number_of_clusters: upper range for cluster computation, minimum set for 2, cannot exceed invariants number

library required:

cluster

```
veni1 <- function(distance_metric,joining_method,path,invariants_input_file,number_of_clusters)
{
  file_path = paste(path, invariants_input_file, sep = "")
  plik=read.csv(file=file_path, sep=";")
  plik=plik[,-1]
  distance=dist(plik,method=distance_metric)
  clustersMatrix=hclust(distance,method=joining_method)
  file_path = paste(path,joining_method,"_",distance_metric,"_","dendrogram.pdf",sep="")
  pdf(file_path, width=170, height=170, bg = "white")
  plot(clustersMatrix,hang=-1)
  dev.off()
  file_path = paste(path,joining_method,"_",distance_metric,"_","clusters",".pdf",sep="")
  pdf(file_path, width=10, height=200, bg = "white")
  file_path = paste(path,joining_method,"_",distance_metric,"_","clusters",".txt",sep="")

  for(i in 2:number_of_clusters)
  {
        sil <- silhouette(cutree(clustersMatrix,i),distance)
        sortSilhouette(sil)
     plot(silhouette(cutree(clustersMatrix,i),distance), cex.names = 0.5)
     #cat(paste(summary(sil)), file_path, append=TRUE)
        print(summary(sil))
  }
  dev.off()
}
```

Script 2:
parameters:

distance_metric: *pearson, correlation*
joining _method: *average, centroid, complete, mcquitty, median, single, ward*
path: path for invariants file
invariants_input_file: name of invariants file
number_of_clusters: upper range for cluster computation, minimum set for 2, cannot exceed invariants number

library required:

cluster
amap

```
veni2 <- function(distance_metric,joining_method,path,invariants_input_file,number_of_clusters)
{
  file_path = paste(path, invariants_input_file, sep = "")
  plik=read.csv(file=file_path, sep=";")
  plik=plik[,-1]
  distance=Dist(plik,method=distance_metric)
  clustersMatrix=hcluster(plik,method=distance_metric,link=joining_method)
  file_path = paste(path,joining_method,"_",distance_metric,"_","dendrogram.pdf",sep="")
  pdf(file_path, width=170, height=170, bg = "white")
  plot(clustersMatrix,hang=-1)
  dev.off()
  file_path = paste(path,joining_method,"_",distance_metric,"_","clusters",".pdf",sep="")
  pdf(file_path, width=10, height=200, bg = "white")
  for(i in 2:number_of_clusters)
  {
    sil <- silhouette(cutree(clustersMatrix,i),distance)
    sortSilhouette(sil)
    plot(silhouette(cutree(clustersMatrix,i),distance))
    print(summary(sil))
  }
  dev.off()
}
```

Example of invariants file in the desired csv format:

first line: names of transition

second and next: t-invariants as a vector, in each line first number is the index of the t-invariants counting from 1.

;0.HIF_1_alpha_SH_and_HIF_1_beta_dimerization;1.running_genes_responsible_for_cell_adaptation_to_hypoxia;2.binding_VEGR_and_VEGFR_2;3.VEGFR2_expression;4.starting_a_tyrosine_kinase_signalling_cascade;5.GLUT1_induced_processes;6.binding_EPO_and_EPOR;7.constitutively_expression_of_HIF_1_beta;8.hydroxylation_of_proline;9.conservative_hydrolysis_of_asparagine;10.binding_HIF_1_OH_OH_and_VHL_VBP_1;11.no_HIF_1_alpha_HIF_1_beta_dimerization;12.low_concentration_of_oxygen_in_the_organism;13.HIF_1_hydroxylases_inactivation;14.CBP_p300_synthesis;15.no_degradation_of_HIF_1_alpha;16.normalization_of_oxygen_status_in_the_organism;17.VHL_synthesis;18.NO_synthesis;19.MMPS_pericytes_recruitment_and_invasion;20.PDGF_AB_and_BB_endothelial_secretion_by_pericytes;21.FGFRs_activation;22.PDGFRs_upregulation;23.binding_PDGFAB_and_BB_with_PDGFRs_activated;24.removal_of_ligands;25.pericytes_and_smooth_muscles_cells_migration;26.strong_endothelial_activation;27.endothelial_proliferation_and_migration;28.binding;29.new_stabilizated_vessels;30.eNOS_activation_by_interraction_with_CaM_Ca_complex;31.cleavage;32.caveolin_usage;33.processes_increasing_shear_stress;34.CaM_caveolin_1_binding;35.caveolin_1_synthesized_in_ER;36.binding_angiopoietins_with_tie_receptor_on_endothelial_cells;37.constitutively_expression_of_angiopoietin_1;38.Tie_expression;39.angiopoietin_expression;40.vessels_remodelling;41.S1P_and_EDG1_binding;42.S1P_synthesis;43.EDG_1_synthesis;44.ECM_enhancing_and_increasing_synthesis_and_release_of_HB_EGF;45.binding_HB_EGF_with_EGFR;46.TGFbeta1_TGFbetaRII_binding;47.activin_like_kinase_5_ALK_5_activation;48.TGFbetaRII_synthesis;49.TGFbeta1_synthesis;50.complex_binding_to_HREs_hypoxia_responsive_elements;51.synthesis_factors_that_variously_stimulate_vessels_permability;52.synthesis_factors_that_variously_stimulate_proliferation_survival_bFGF;53.synthesis_factors_that_variously_stimulate_migration_MMPs;54.increase_in_intracellular_Ca;55.CaM_EPOR_binding;56.upregulation_of_EPOR_signalling_pathways;57.CaM_binds_Ca;58.CaM_synthesis;59.HIF_2_alpha_influence_on_EPO;60.Sirtuin_1_influence_on_HIF_2_alpha;61.Sirtuin_1_synthesis;62.Fe_2_increasing;63.HIF_1_alpha_asparagine_hydroxylases_activation;64.HIF_1_al

pha_proline_hydroxylases_ativation;65.oxygenation;66.activation_of_proteasome_degradation;67.ubiquitin_synthesis;
68.BTM_synthesis;69.S_nitrosylation;70.increasing_activity;71.processess_increasing_NO;72.EPOR_synthesis_induced_
by_hypoxia;73.normal_state
1;0;0;0;0;0;0;0;0;0;0;0;0;0;0;0;0;0;0;0;0;0;0;0;0;0;0;0;0;0;0;1;0;0;1;0;0;0;0;0;0;0;0;0;0;0;0;0;0;0;0;0;0;0;0;0;0;0;0;0;0;
0;0;0;0;0;0;0;0;0;0;0;0
2;0;0;0;0;0;0;0;0;0;0;0;0;0;0;0;0;0;0;0;0;0;0;0;0;0;0;0;0;0;0;0;0;0;0;0;0;0;0;0;0;0;0;0;0;0;1;1;1;1;0;0;0;0;0;0;0;0;0;0;0;0;
0;0;0;0;0;0;0;0;0;0;0;0
3;0;0;0;0;0;0;0;0;0;0;0;0;0;0;0;0;0;0;0;0;0;0;0;0;0;0;0;1;0;0;0;0;0;0;0;0;0;1;1;0;0;0;0;0;0;0;0;0;0;0;0;0;0;0;0;0;0;0;1;1;
0;0;0;0;0;0;0;0;0;0;0;0
4;0;0;0;0;0;0;0;0;0;0;0;0;0;0;0;0;0;0;0;0;0;0;0;0;0;0;0;0;0;1;0;1;1;0;0;0;0;0;0;0;0;0;0;0;0;0;0;0;0;0;0;0;0;0;0;0;0;1;1;1;
0;0;0;0;0;0;0;0;0;0;0;0
5;0;0;0;0;0;0;0;1;1;1;1;0;0;0;0;1;0;0;0;0;0;0;0;0;0;0;0;0;0;0;0;0;0;0;0;0;0;0;0;0;0;0;0;0;0;0;0;0;0;0;0;0;0;0;0;0;0;0;0;0;
1;1;1;1;1;1;0;0;0;0;0;1
6;1;1;1;1;1;1;0;1;0;0;0;0;1;0;1;0;0;0;1;0;1;0;0;0;0;0;0;0;1;0;0;0;0;0;0;0;0;0;0;0;0;0;0;0;0;0;0;0;1;1;0;0;1;0;0;1;1;0;0;0;
0;0;0;0;0;0;1;1;0;0;0;1
7;0;0;0;0;0;0;0;1;1;1;1;1;0;0;0;1;1;0;0;0;0;0;0;0;0;0;0;0;0;0;0;0;0;0;0;0;0;0;0;0;0;0;0;0;0;0;0;0;0;0;0;0;0;0;0;0;0;0;0;0;
1;1;1;1;1;1;0;0;0;0;0;0
8;1;1;1;1;1;1;0;1;0;0;0;0;1;0;1;0;1;0;1;0;1;0;0;0;0;0;0;0;1;0;0;0;0;0;0;0;0;0;0;0;0;0;0;0;0;0;0;0;1;1;0;0;1;0;0;1;1;0;0;0;
0;0;0;1;0;0;1;1;0;0;0;0
9;0;0;0;0;0;0;0;1;1;1;0;0;0;0;0;1;0;0;0;0;0;0;0;0;0;0;0;0;0;0;0;0;0;0;0;0;0;0;0;0;0;0;0;0;0;0;0;0;0;0;0;0;0;0;0;0;0;0;0;0;
1;1;0;1;1;1;0;0;1;1;0;1
10;1;1;1;1;1;1;0;1;0;0;0;0;1;0;1;0;0;0;1;0;1;0;0;0;0;0;0;0;0;0;1;0;1;1;0;0;0;0;0;0;0;0;0;0;0;0;0;0;1;1;0;0;0;0;0;0;0;0;0;0
;0;0;0;0;0;0;1;1;0;1;0;1
11;1;1;1;1;1;1;0;1;0;0;0;0;1;0;1;0;0;0;0;1;1;1;1;1;1;0;1;1;1;0;1;0;1;1;0;1;1;1;1;0;1;1;1;1;1;1;1;0;1;0;1;1;0;0;0;0;0;0;1;1
;0;0;0;0;0;0;1;1;0;1;0;1
12;1;1;1;1;1;1;1;1;0;0;0;0;1;0;1;0;0;0;0;1;1;1;1;1;1;0;0;1;1;0;1;0;1;1;0;1;1;1;1;0;1;1;1;1;1;1;1;0;1;0;1;1;1;1;1;1;1;0;1;1
;0;0;0;0;0;0;1;1;0;1;1;1
13;1;1;1;1;1;1;0;1;0;0;0;0;1;1;1;1;0;0;1;0;1;0;0;0;0;1;0;0;0;0;1;0;1;1;0;0;0;0;0;0;0;0;0;0;0;0;0;0;1;1;0;0;0;0;0;0;0;0;0;0
;0;0;0;0;0;0;1;0;0;1;0;1
14;1;1;1;1;1;1;0;1;0;0;0;0;1;1;1;1;0;0;0;1;1;1;1;1;1;1;1;1;1;1;0;1;0;1;1;0;1;1;1;1;0;1;1;1;1;1;1;1;0;1;0;1;1;0;0;0;0;0;0;1;1
;0;0;0;0;0;0;1;0;0;1;0;1
15;1;1;1;1;1;1;1;1;0;0;0;0;1;1;1;1;0;0;0;1;1;1;1;1;1;1;0;1;1;0;1;0;1;1;0;1;1;1;1;0;1;1;1;1;1;1;1;0;1;0;1;1;1;1;1;1;1;0;1;1
;0;0;0;0;0;0;1;0;0;1;1;1
16;1;1;1;1;1;1;0;1;0;0;0;0;1;0;1;0;0;0;1;1;1;1;1;1;1;0;1;1;1;1;0;0;0;0;1;1;1;1;0;1;1;1;1;1;1;1;0;1;0;1;1;1;0;0;1;1;0;1;1
;0;0;0;0;0;0;1;1;0;1;0;1
17;1;1;1;1;1;1;1;1;0;0;0;0;1;0;1;0;0;0;1;1;1;1;1;1;1;0;0;1;1;1;0;0;0;0;1;1;1;1;0;1;1;1;1;1;1;1;0;1;0;1;1;1;1;1;1;1;0;1;1
;0;0;0;0;0;0;1;1;0;1;1;1
18;1;1;1;1;1;1;0;1;0;0;0;0;1;1;1;1;0;0;1;1;1;1;1;1;1;1;1;1;1;1;0;0;0;0;1;1;1;1;0;1;1;1;1;1;1;1;0;1;0;1;1;1;0;0;1;1;0;1;1
;0;0;0;0;0;0;1;0;0;1;0;1
19;1;1;1;1;1;1;1;1;0;0;0;0;1;1;1;1;0;0;1;1;1;1;1;1;1;1;0;1;1;1;0;0;0;0;1;1;1;1;0;1;1;1;1;1;1;1;0;1;0;1;1;1;1;1;1;1;0;1;1
;0;0;0;0;0;0;1;0;0;1;1;1
20;0;0;0;0;0;0;0;1;1;1;1;1;0;0;0;1;1;0;0;0;0;0;0;0;0;0;0;0;0;0;0;0;0;0;0;0;0;0;0;0;0;0;0;0;0;0;0;0;0;0;0;0;0;0;0;0;0;0;0;0
;1;1;0;1;1;1;0;0;1;1;0;0
21;1;1;1;1;1;1;0;1;0;0;0;0;1;0;1;0;1;0;1;0;1;0;0;0;0;0;0;0;0;0;1;0;1;1;0;0;0;0;0;0;0;0;0;0;0;0;0;0;1;1;0;0;0;0;0;0;0;0;0;0
;0;0;0;1;0;0;1;1;0;1;0;0
22;1;1;1;1;1;1;0;1;0;0;0;0;1;0;1;0;1;0;0;1;1;1;1;1;1;0;1;1;1;0;1;0;1;1;0;1;1;1;1;0;1;1;1;1;1;1;1;0;1;0;1;1;0;0;0;0;0;0;1;1
;0;0;0;1;0;0;1;1;0;1;0;0
23;1;1;1;1;1;1;1;1;0;0;0;0;1;0;1;0;1;0;0;1;1;1;1;1;1;0;0;1;1;0;1;0;1;1;0;1;1;1;1;0;1;1;1;1;1;1;1;0;1;0;1;1;1;1;1;1;1;0;1;1
;0;0;0;1;0;0;1;1;0;1;1;0
24;1;1;1;1;1;1;0;1;0;0;0;0;1;1;1;1;1;0;1;0;1;0;0;0;0;1;0;0;0;0;1;0;1;1;0;0;0;0;0;0;0;0;0;0;0;0;0;0;1;1;0;0;0;0;0;0;0;0;0;0
;0;0;0;1;0;0;1;0;0;1;0;0
25;1;1;1;1;1;1;0;1;0;0;0;0;1;1;1;1;1;0;0;1;1;1;1;1;1;1;1;1;1;1;0;1;0;1;1;0;1;1;1;1;0;1;1;1;1;1;1;1;0;1;0;1;1;0;0;0;0;0;0;1;1
;0;0;0;1;0;0;1;0;0;1;0;0

26;1;1;1;1;1;1;1;1;0;0;0;0;1;1;1;1;1;0;0;1;1;1;1;1;1;1;0;1;1;0;1;0;1;1;0;1;1;1;1;0;1;1;1;1;1;1;1;0;1;0;1;1;1;1;1;1;1;0;1;1
;0;0;0;1;0;0;1;0;0;1;1;0
27;1;1;1;1;1;1;0;1;0;0;0;0;1;0;1;0;1;0;1;1;1;1;1;1;1;1;0;1;1;1;1;0;0;0;0;0;1;1;1;1;0;1;1;1;1;1;1;1;0;1;0;1;1;1;0;0;1;1;0;1;1
;0;0;0;1;0;0;1;1;0;1;0;0
28;1;1;1;1;1;1;1;1;0;0;0;0;1;0;1;0;1;0;1;1;1;1;1;1;1;0;0;1;1;1;0;0;0;0;0;1;1;1;1;0;1;1;1;1;1;1;1;0;1;0;1;1;1;1;1;1;1;0;1;1
;0;0;0;1;0;0;1;1;0;1;1;0
29;1;1;1;1;1;1;0;1;0;0;0;0;1;1;1;1;1;0;1;1;1;1;1;1;1;1;1;1;1;1;0;0;0;0;0;1;1;1;1;0;1;1;1;1;1;1;1;0;1;0;1;1;1;0;0;1;1;0;1;1
;0;0;0;1;0;0;1;0;0;1;0;0
30;1;1;1;1;1;1;1;1;0;0;0;0;1;1;1;1;1;0;1;1;1;1;1;1;1;1;0;1;1;1;0;0;0;0;0;1;1;1;1;0;1;1;1;1;1;1;1;0;1;0;1;1;1;1;1;1;1;0;1;1
;0;0;0;1;0;0;1;0;0;1;1;0
31;0;0;0;0;0;0;0;0;1;1;1;1;0;0;0;0;0;1;1;0;0;0;0;0;0;0;0;0;0;1;0;0;0;0;0;0;0;0;0;0;0;0;0;0;0;0;0;0;0;0;0;0;1;0;0;1;1;1;1;1
;1;1;0;1;1;1;0;0;1;0;0;1
32;1;1;1;1;1;1;0;1;0;0;0;0;1;0;1;0;0;0;1;1;1;1;1;1;1;0;1;1;1;1;0;0;0;0;0;1;1;1;1;0;1;1;1;1;1;1;1;0;1;0;1;1;1;0;0;1;1;1;1;1
;0;0;0;0;0;0;1;1;0;0;0;1
33;1;1;1;1;1;1;1;1;0;0;0;0;1;0;1;0;0;0;1;1;1;1;1;1;1;0;0;1;1;1;0;0;0;0;0;1;1;1;1;0;1;1;1;1;1;1;1;0;1;0;1;1;1;1;1;1;1;1;1;1
;0;0;0;0;0;0;1;1;0;0;1;1
34;1;1;1;1;1;1;0;1;0;0;0;0;1;1;1;1;0;0;1;1;1;1;1;1;1;1;1;1;1;1;0;0;0;0;0;1;1;1;1;0;1;1;1;1;1;1;1;0;1;0;1;1;1;0;0;1;1;1;1;1
;0;0;0;0;0;0;1;0;0;0;0;1
35;1;1;1;1;1;1;1;1;0;0;0;0;1;1;1;1;0;0;1;1;1;1;1;1;1;1;0;1;1;1;0;0;0;0;0;1;1;1;1;0;1;1;1;1;1;1;1;0;1;0;1;1;1;1;1;1;1;1;1;1
;0;0;0;0;0;0;1;0;0;0;1;1
36;0;0;0;0;0;0;0;0;1;1;1;1;1;0;0;0;1;1;1;0;0;0;0;0;0;0;0;0;0;1;0;0;0;0;0;0;0;0;0;0;0;0;0;0;0;0;0;0;0;0;0;0;1;0;0;1;1;1;1;1
;1;1;0;1;1;1;0;0;1;0;0;0
37;1;1;1;1;1;1;0;1;0;0;0;0;1;0;1;0;1;0;1;1;1;1;1;1;1;1;0;1;1;1;1;0;0;0;0;0;1;1;1;1;0;1;1;1;1;1;1;1;0;1;0;1;1;1;0;0;1;1;1;1;1
;0;0;0;1;0;0;1;1;0;0;0;0
38;1;1;1;1;1;1;1;1;0;0;0;0;1;0;1;0;1;0;1;1;1;1;1;1;1;0;0;1;1;1;0;0;0;0;0;1;1;1;1;0;1;1;1;1;1;1;1;0;1;0;1;1;1;1;1;1;1;1;1;1
;0;0;0;1;0;0;1;1;0;0;1;0
39;1;1;1;1;1;1;0;1;0;0;0;0;1;1;1;1;1;0;1;1;1;1;1;1;1;1;1;1;1;1;0;0;0;0;0;1;1;1;1;0;1;1;1;1;1;1;1;0;1;0;1;1;1;0;0;1;1;1;1;1
;0;0;0;1;0;0;1;0;0;0;0;0
40;1;1;1;1;1;1;1;1;0;0;0;0;1;1;1;1;1;0;1;1;1;1;1;1;1;1;0;1;1;1;0;0;0;0;0;1;1;1;1;0;1;1;1;1;1;1;1;0;1;0;1;1;1;1;1;1;1;1;1;1
;0;0;0;1;0;0;1;0;0;0;1;0
41;1;1;1;1;1;1;0;1;1;1;1;1;1;1;1;1;0;1;1;0;1;0;0;0;0;0;1;0;0;0;1;0;0;0;0;0;0;0;0;0;0;0;0;0;0;0;0;0;1;1;0;0;1;0;0;1;1;0;0;0
;1;1;0;1;1;1;1;0;1;0;0;1
42;1;1;1;1;1;1;0;1;0;0;0;0;1;1;1;1;0;0;1;0;1;0;0;0;0;0;1;0;0;0;1;1;0;1;1;0;0;0;0;0;0;0;0;0;0;0;0;0;1;1;0;0;1;0;0;1;1;0;0;0
;0;0;0;0;0;0;1;0;0;0;0;1
43;1;1;1;1;1;1;0;1;0;0;0;0;1;1;1;1;0;0;1;1;1;1;1;1;1;1;1;1;1;1;0;0;0;0;0;1;1;1;1;0;1;1;1;1;1;1;1;0;1;1;1;1;1;0;0;1;1;0;1;1
;0;0;0;0;0;0;1;0;0;0;0;1
44;1;1;1;1;1;1;1;1;0;0;0;0;1;1;1;1;0;0;1;1;1;1;1;1;1;1;0;1;1;1;0;0;0;0;0;1;1;1;1;0;1;1;1;1;1;1;1;0;1;1;1;1;1;1;1;1;1;0;1;1
;0;0;0;0;0;0;1;0;0;0;1;1
45;1;1;1;1;1;1;0;1;1;1;1;1;1;1;1;1;1;1;0;1;0;0;0;0;0;1;0;0;0;1;0;0;0;0;0;0;0;0;0;0;0;0;0;0;0;0;0;0;1;1;0;0;1;0;0;1;1;0;0;0
;1;1;0;1;1;1;1;0;1;0;0;0
46;1;1;1;1;1;1;0;1;0;0;0;0;1;1;1;1;1;0;1;0;1;0;0;0;0;0;1;0;0;0;1;1;0;1;1;0;0;0;0;0;0;0;0;0;0;0;0;0;1;1;0;0;1;0;0;1;1;0;0;0
;0;0;0;1;0;0;1;0;0;0;0;0
47;1;1;1;1;1;1;0;1;0;0;0;0;1;1;1;1;1;0;1;1;1;1;1;1;1;1;1;1;1;1;0;0;0;0;0;1;1;1;1;0;1;1;1;1;1;1;1;0;1;1;1;1;1;0;0;1;1;0;1;1
;0;0;0;1;0;0;1;0;0;0;0;0
48;1;1;1;1;1;1;1;1;0;0;0;0;1;1;1;1;1;0;1;1;1;1;1;1;1;1;0;1;1;1;0;0;0;0;0;1;1;1;1;0;1;1;1;1;1;1;1;0;1;1;1;1;1;1;1;1;1;0;1;1
;0;0;0;1;0;0;1;0;0;0;1;0
