# Supplementary material for: Petri net-based approach to modeling and analysis of selected aspects of the molecular regulation of angiogenesis
Source: PLoS One. 2017 Mar 2;12(3):e0173020. doi: 10.1371/journal.pone.0173020 (PMC5333880; doi:10.1371/journal.pone.0173020)
Supplement: S2 Table — (PDF) [file pone.0173020.s003.pdf]

S2 Table. Knockout results for all transitions when transition t_1 has been disabled

**Transition**	**Transition status**	**Reference set**	**Knockout set**	**Difference**
t_0	ACTIVE	14.98%	11.17%	−3.82%
t_1	OFFLINE	12.62%	0.00%	disabled
t_2	KNOCKOUT	12.62%	0.00%	disabled
t_3	ACTIVE	50.00%	50.00%	0.00%
t_4	KNOCKOUT	12.62%	0.00%	disabled
t_5	KNOCKOUT	12.48%	0.00%	disabled
t_6	ACTIVE	2.35%	8.00%	5.65%
t_7	ACTIVE	14.99%	11.17%	−3.82%
t_8	ACTIVE	34.71%	36.03%	1.32%
t_9	ACTIVE	21.24%	22.19%	0.95%
t_{10}	ACTIVE	34.69%	36.01%	1.32%
t_{11}	ACTIVE	21.23%	22.18%	0.95%
t_{12}	ACTIVE	50.00%	49.99%	−0.01%
t_{13}	ACTIVE	22.80%	17.24%	−5.57%
t_{14}	ACTIVE	50.01%	50.00%	−0.01%
t_{15}	ACTIVE	13.46%	13.83%	0.37%
t_{16}	ACTIVE	7.49%	5.59%	−1.91%
t_{17}	ACTIVE	49.99%	49.99%	0.00%
t_{18}	ACTIVE	16.39%	5.43%	−10.96%
t_{19}	KNOCKOUT	4.20%	0.00%	disabled
t_{20}	ACTIVE	50.01%	50.01%	0.00%
t_{21}	KNOCKOUT	4.21%	0.00%	disabled
t_{22}	KNOCKOUT	4.20%	0.00%	disabled
t_{23}	KNOCKOUT	4.20%	0.00%	disabled
t_{24}	KNOCKOUT	4.20%	0.00%	disabled
t_{25}	KNOCKOUT	4.09%	0.00%	disabled
t_{26}	ACTIVE	22.81%	17.24%	−5.57%
t_{27}	ACTIVE	13.48%	13.86%	0.38%
t_{28}	KNOCKOUT	4.07%	0.00%	disabled
t_{29}	ACTIVE	16.42%	12.36%	−4.06%
t_{30}	ACTIVE	12.19%	5.43%	−6.76%
t_{31}	ACTIVE	20.94%	15.85%	−5.09%
t_{32}	ACTIVE	43.53%	47.42%	3.89%
t_{33}	ACTIVE	50.02%	50.00%	−0.02%
t_{34}	ACTIVE	27.37%	18.32%	−9.05%
t_{35}	ACTIVE	50.00%	50.01%	0.01%
t_{36}	ACTIVE	12.35%	12.34%	−0.01%
t_{37}	ACTIVE	21.00%	15.91%	−5.09%
t_{38}	ACTIVE	49.99%	49.99%	0.00%
t_{39}	ACTIVE	24.71%	24.71%	0.00%
t_{40}	ACTIVE	12.36%	12.37%	0.01%
t_{41}	ACTIVE	49.27%	49.27%	0.00%
t_{42}	ACTIVE	49.99%	50.00%	0.01%
t_{43}	ACTIVE	50.00%	50.01%	0.01%
t_{44}	ACTIVE	8.28%	12.34%	4.06%
t_{45}	KNOCKOUT	4.20%	0.00%	disabled
t_{46}	ACTIVE	49.43%	49.27%	−0.16%
t_{47}	ACTIVE	49.04%	48.92%	−0.12%
t_{48}	ACTIVE	50.00%	49.99%	0.00%
t_{49}	ACTIVE	49.99%	50.00%	0.01%
t_{50}	ACTIVE	14.98%	11.16%	−3.82%
t_{51}	KNOCKOUT	4.20%	0.00%	disabled
t_{52}	KNOCKOUT	4.21%	0.00%	disabled
t_{53}	KNOCKOUT	4.20%	0.00%	disabled
t_{54}	ACTIVE	20.99%	15.91%	−5.09%
t_{55}	ACTIVE	2.35%	7.99%	5.64%
t_{56}	ACTIVE	2.35%	7.98%	5.63%
t_{57}	ACTIVE	20.99%	15.90%	−5.09%
t_{58}	ACTIVE	50.01%	50.00%	−0.01%
t_{59}	ACTIVE	24.71%	24.71%	0.00%

Transition	Transition status	Reference set	Knockout set	Difference
t_{60}	ACTIVE	49.43%	49.43%	0.00%
t_{61}	ACTIVE	50.00%	49.99%	−0.01%
t_{62}	ACTIVE	50.00%	50.01%	0.01%
t_{63}	ACTIVE	21.24%	22.19%	0.95%
t_{64}	ACTIVE	21.24%	22.18%	0.94%
t_{65}	ACTIVE	50.00%	49.99%	0.00%
t_{66}	ACTIVE	34.68%	35.99%	1.31%
t_{67}	ACTIVE	50.00%	50.01%	0.01%
t_{68}	ACTIVE	50.01%	50.00%	0.00%
t_{69}	ACTIVE	13.48%	13.86%	0.39%
t_{70}	ACTIVE	13.48%	13.86%	0.38%
t_{71}	ACTIVE	50.00%	49.99%	0.00%
t_{72}	ACTIVE	2.35%	8.01%	5.66%
t_{73}	ACTIVE	50.00%	49.99%	−0.01%
