# Supplementary material for: Petri net-based approach to modeling and analysis of selected aspects of the molecular regulation of angiogenesis
Source: PLoS One. 2017 Mar 2;12(3):e0173020. doi: 10.1371/journal.pone.0173020 (PMC5333880; doi:10.1371/journal.pone.0173020)
Supplement: S3 Table — (PDF) [file pone.0173020.s004.pdf]

S3 Table. Knockout results for all transitions when transitions t_{12}, t_{18} and MCT-set m_2 have been disabled

**Transition**	**Transition status**	**Reference set**	**Knockout set**	**Difference**
t_0	OFFLINE (MCT2)	14.98%	0.00%	disabled
t_1	OFFLINE (MCT2)	12.62%	0.00%	disabled
t_2	OFFLINE (MCT2)	12.62%	0.00%	disabled
t_3	OFFLINE (MCT2)	50.00%	0.00%	disabled
t_4	OFFLINE (MCT2)	12.62%	0.00%	disabled
t_5	OFFLINE (MCT2)	12.48%	0.00%	disabled
t_6	KNOCKOUT	2.35%	0.00%	disabled
t_7	OFFLINE (MCT2)	14.99%	0.00%	disabled
t_8	ACTIVE	34.71%	41.40%	6.69%
t_9	ACTIVE	21.24%	24.78%	3.55%
t_{10}	ACTIVE	34.69%	41.37%	6.67%
t_{11}	ACTIVE	21.23%	24.78%	3.54%
t_{12}	OFFLINE	50.00%	0.00%	disabled
t_{13}	KNOCKOUT	22.80%	0.00%	disabled
t_{14}	OFFLINE (MCT2)	50.01%	0.00%	disabled
t_{15}	KNOCKOUT	13.46%	0.00%	disabled
t_{16}	KNOCKOUT	7.49%	0.00%	disabled
t_{17}	ACTIVE	49.99%	49.99%	0.00%
t_{18}	OFFLINE	16.39%	0.00%	disabled
t_{19}	KNOCKOUT	4.20%	0.00%	disabled
t_{20}	OFFLINE (MCT2)	50.01%	0.00%	disabled
t_{21}	KNOCKOUT	4.21%	0.00%	disabled
t_{22}	KNOCKOUT	4.20%	0.00%	disabled
t_{23}	KNOCKOUT	4.20%	0.00%	disabled
t_{24}	KNOCKOUT	4.20%	0.00%	disabled
t_{25}	KNOCKOUT	4.09%	0.00%	disabled
t_{26}	KNOCKOUT	22.81%	0.00%	disabled
t_{27}	ACTIVE	13.48%	16.67%	3.19%
t_{28}	KNOCKOUT	4.07%	0.00%	disabled
t_{29}	ACTIVE	16.42%	17.07%	0.65%
t_{30}	ACTIVE	12.19%	1.79%	−10.40%
t_{31}	ACTIVE	20.94%	7.63%	−13.31%
t_{32}	ACTIVE	43.53%	44.11%	0.58%
t_{33}	ACTIVE	50.02%	50.03%	0.00%
t_{34}	ACTIVE	27.37%	13.48%	−13.89%
t_{35}	ACTIVE	50.00%	50.00%	0.00%
t_{36}	ACTIVE	12.35%	7.63%	−4.72%
t_{37}	ACTIVE	21.00%	7.65%	−13.35%
t_{38}	ACTIVE	49.99%	49.99%	0.00%
t_{39}	ACTIVE	24.71%	24.71%	0.00%
t_{40}	ACTIVE	12.36%	17.07%	4.71%
t_{41}	ACTIVE	49.27%	49.27%	−0.01%
t_{42}	ACTIVE	49.99%	50.00%	0.01%
t_{43}	ACTIVE	50.00%	50.00%	0.00%
t_{44}	ACTIVE	8.28%	7.63%	−0.65%
t_{45}	KNOCKOUT	4.20%	0.00%	disabled
t_{46}	ACTIVE	49.43%	49.27%	−0.16%
t_{47}	ACTIVE	49.04%	48.92%	−0.12%
t_{48}	ACTIVE	50.00%	50.01%	0.01%
t_{49}	ACTIVE	49.99%	50.00%	0.01%
t_{50}	OFFLINE (MCT2)	14.98%	0.00%	disabled
t_{51}	KNOCKOUT	4.20%	0.00%	disabled
t_{52}	KNOCKOUT	4.21%	0.00%	disabled
t_{53}	KNOCKOUT	4.20%	0.00%	disabled
t_{54}	ACTIVE	20.99%	7.65%	−13.34%
t_{55}	KNOCKOUT	2.35%	0.00%	disabled
t_{56}	KNOCKOUT	2.35%	0.00%	disabled
t_{57}	ACTIVE	20.99%	7.65%	−13.34%
t_{58}	ACTIVE	50.01%	50.01%	0.00%
t_{59}	ACTIVE	24.71%	24.73%	0.02%

Transition	Transition status	Reference set	Knockout set	Difference
t_{60}	ACTIVE	49.43%	49.45%	0.01%
t_{61}	ACTIVE	50.00%	50.02%	0.02%
t_{62}	ACTIVE	50.00%	50.00%	0.00%
t_{63}	ACTIVE	21.24%	24.79%	3.55%
t_{64}	ACTIVE	21.24%	24.78%	3.54%
t_{65}	ACTIVE	50.00%	50.01%	0.01%
t_{66}	ACTIVE	34.68%	41.34%	6.66%
t_{67}	ACTIVE	50.00%	50.02%	0.01%
t_{68}	OFFLINE (MCT2)	50.01%	0.00%	disabled
t_{69}	ACTIVE	13.48%	16.67%	3.19%
t_{70}	ACTIVE	13.48%	16.65%	3.16%
t_{71}	ACTIVE	50.00%	49.99%	−0.01%
t_{72}	KNOCKOUT	2.35%	0.00%	disabled
t_{73}	ACTIVE	50.00%	50.01%	0.00%
